# Supplementary material for: Sources of variation and establishment of Russian reference intervals for major hormones and tumor markers
Source: PLoS One. 2021 Jan 7;16(1):e0234284. doi: 10.1371/journal.pone.0234284 (PMC7790266; doi:10.1371/journal.pone.0234284)
Supplement: S1 Fig — Distributions of RVs for all the analytes were shown after subgrouped by sex and age. No secondary exclusion was performed in plotting data. The box in each scattergram represents central 50% range and the vertical bar in the middle represents a median point. On top of each scattergram, the magnitudes of between-sex and between-age variations are shown as SDRsex and SDRage derived separately for males (M) and females (F). (PDF) [file pone.0234284.s002.pdf]

S1 Fig. Sex and age-related changes in RVs of all immunoassay analytes

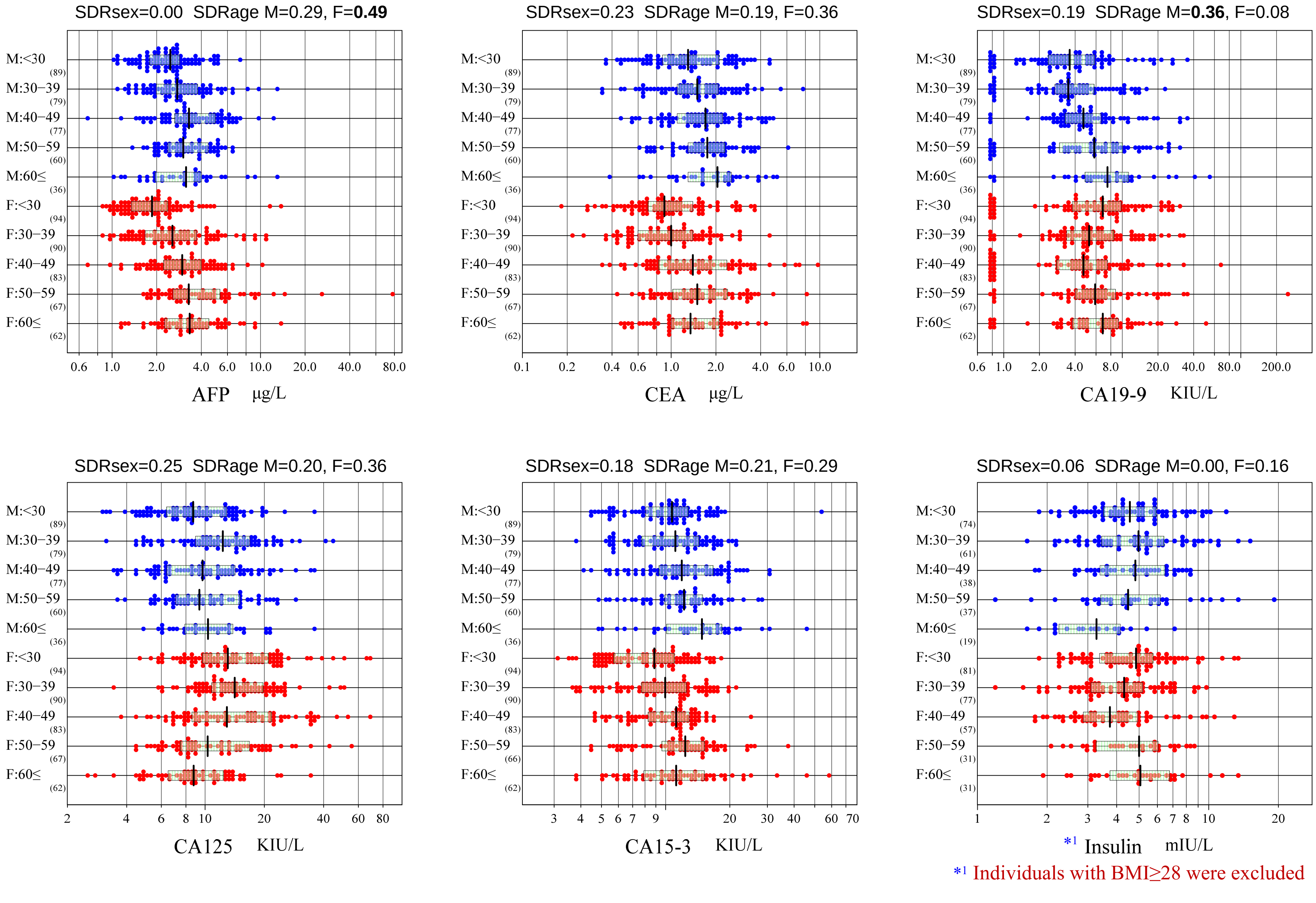

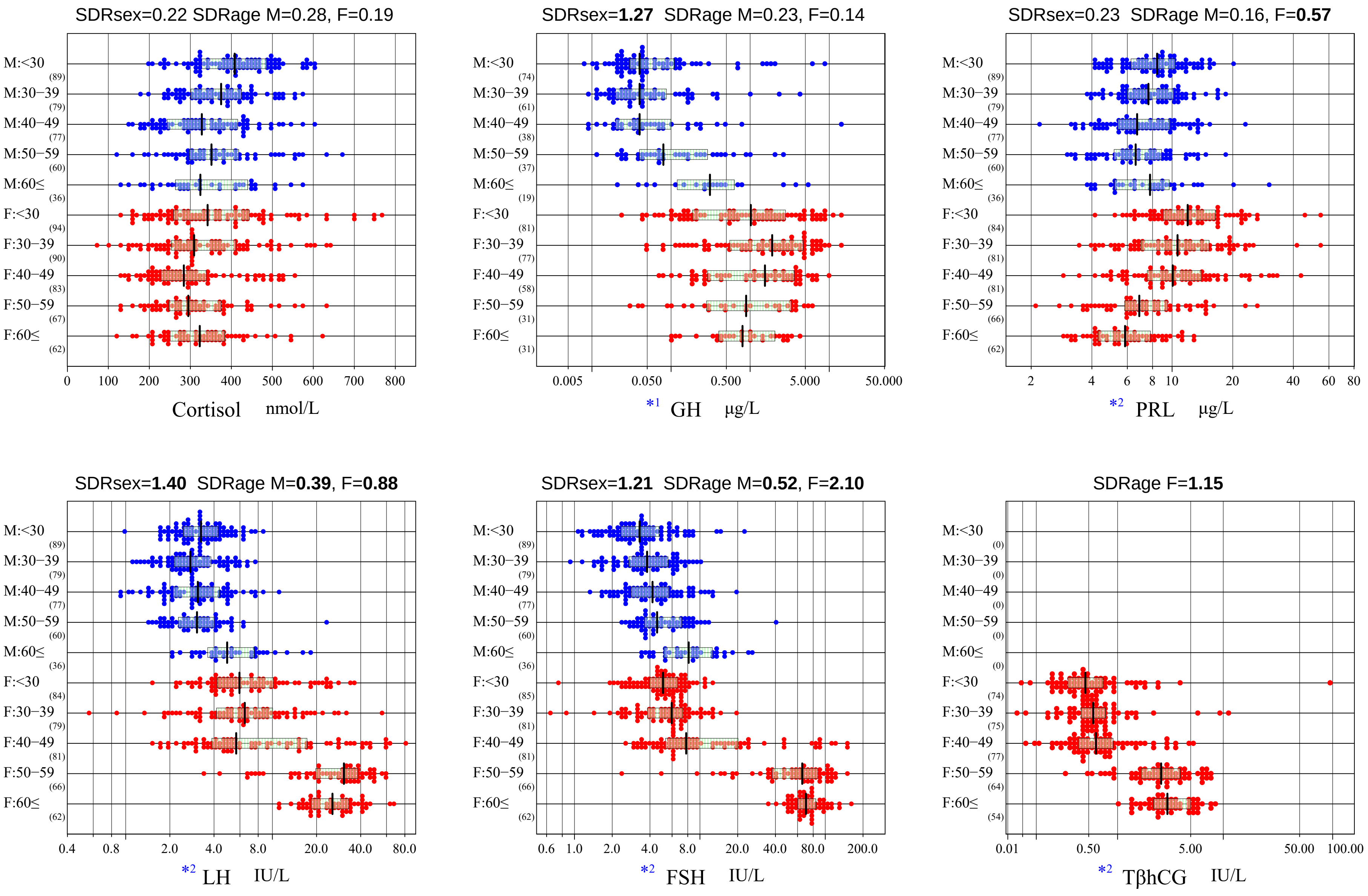

*1 Individuals with BMI≥28 were excluded *2 Individuals with oral contraceptives were excluded

Distributions of RVs for all the analytes were shown after subgrouped by sex and age. No secondary exclusion was performed in plotting data. The box in each scattergram represents central 50% range and the vertical bar in the middle represents a median point. On top of each scattergram, the magnitudes of between-sex and between-age variations are shown as SDRsex and SDRage derived separately for males (M) and females (F).

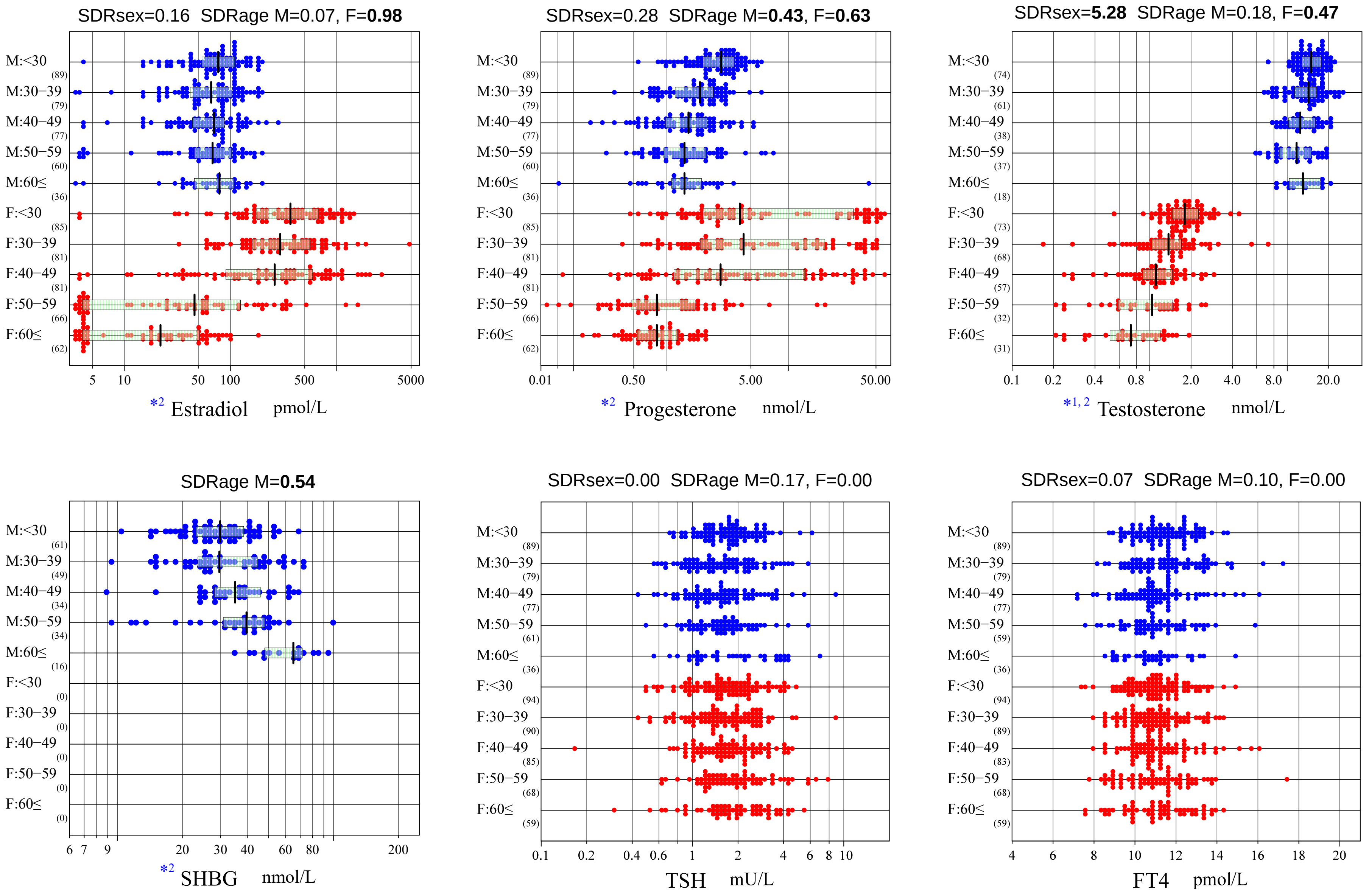

*1 Individuals with BMI≥28 were excluded *2 Individuals with oral contraceptives were excluded

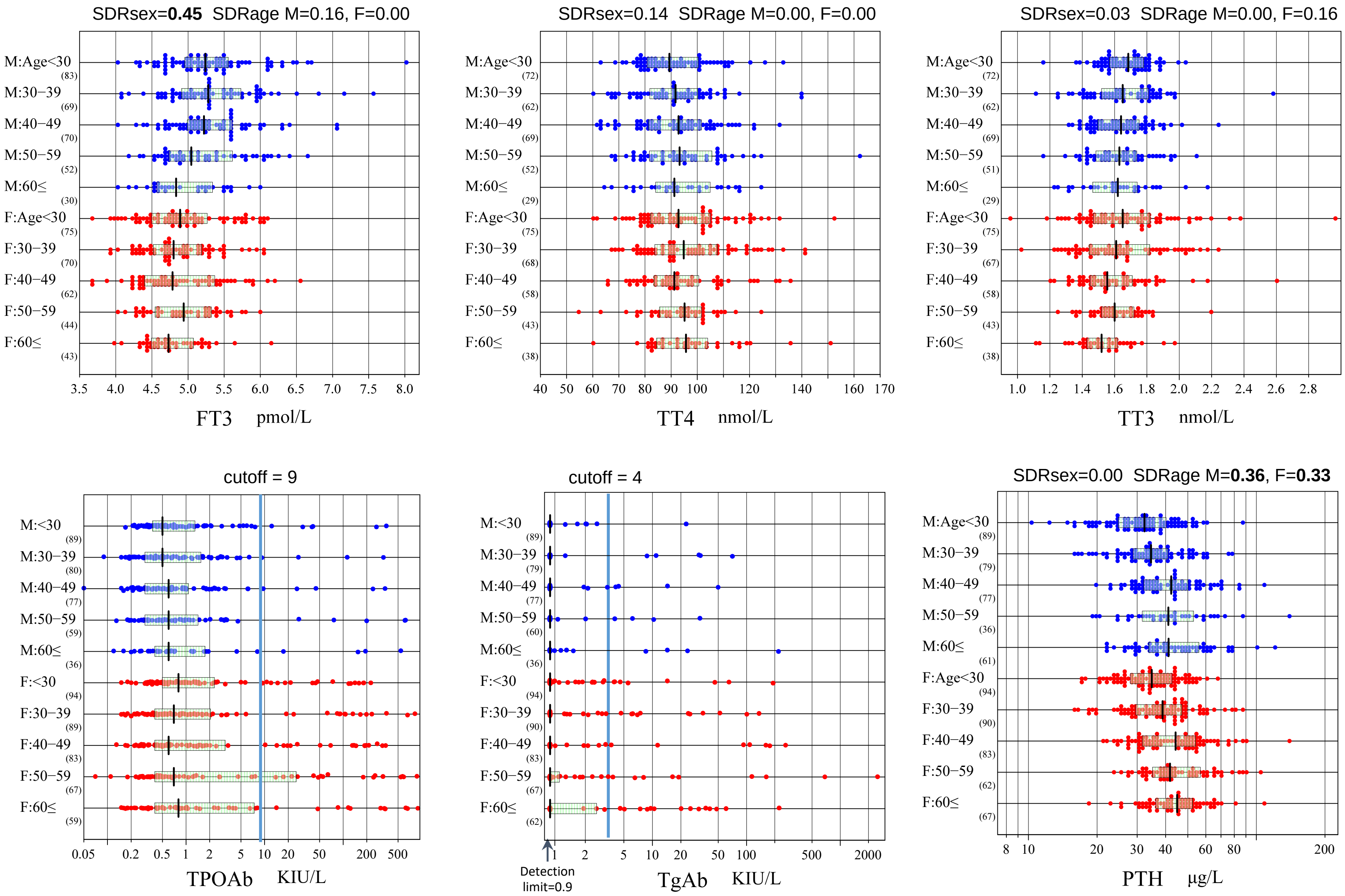

SDRsex=0.45 SDRage M=0.16, F=0.00
M:Age<30 (83)
M:30−39 (69)
M:40−49 (70)
M:50−59 (52)
M:60≤ (30)
F:Age<30 (75)
F:30−39 (70)
F:40−49 (62)
F:50−59 (44)
F:60≤ (43)
3.5 4.0 4.5 5.0 5.5 6.0 6.5 7.0 7.5 8.0
FT3 pmol/L
SDRsex=0.14 SDRage M=0.00, F=0.00
M:Age<30 (72)
M:30−39 (62)
M:40−49 (69)
M:50−59 (52)
M:60≤ (29)
F:Age<30 (75)
F:30−39 (68)
F:40−49 (58)
F:50−59 (43)
F:60≤ (38)
40 50 60 70 80 90 100 120 140 160 170
TT4 nmol/L
SDRsex=0.03 SDRage M=0.00, F=0.16
M:Age<30 (72)
M:30−39 (62)
M:40−49 (69)
M:50−59 (51)
M:60≤ (29)
F:Age<30 (75)
F:30−39 (67)
F:40−49 (58)
F:50−59 (43)
F:60≤ (38)
1.0 1.2 1.4 1.6 1.8 2.0 2.2 2.4 2.6 2.8
TT3 nmol/L
cutoff = 9
M:<30 (89)
M:30−39 (80)
M:40−49 (77)
M:50−59 (59)
M:60≤ (36)
F:<30 (94)
F:30−39 (89)
F:40−49 (83)
F:50−59 (67)
F:60≤ (59)
0.05 0.2 0.5 1 2 5 10 20 50 200 500
TPOAb KIU/L
cutoff = 4
M:<30 (89)
M:30−39 (79)
M:40−49 (77)
M:50−59 (60)
M:60≤ (36)
F:<30 (94)
F:30−39 (90)
F:40−49 (83)
F:50−59 (67)
F:60≤ (62)
1 2 5 10 20 50 100 500 2000
Detection limit=0.9
TgAb KIU/L
SDRsex=0.00 SDRage M=0.36, F=0.33
M:Age<30 (89)
M:30−39 (79)
M:40−49 (77)
M:50−59 (36)
M:60≤ (61)
F:Age<30 (94)
F:30−39 (90)
F:40−49 (83)
F:50−59 (62)
F:60≤ (67)
8 10 20 30 40 60 80 200
PTH μg/L
