# Supplementary material for: Sources of variation and establishment of Russian reference intervals for major hormones and tumor markers
Source: PLoS One. 2021 Jan 7;16(1):e0234284. doi: 10.1371/journal.pone.0234284 (PMC7790266; doi:10.1371/journal.pone.0234284)

S2 Fig. Accuracy of power transformation used in the parametric method

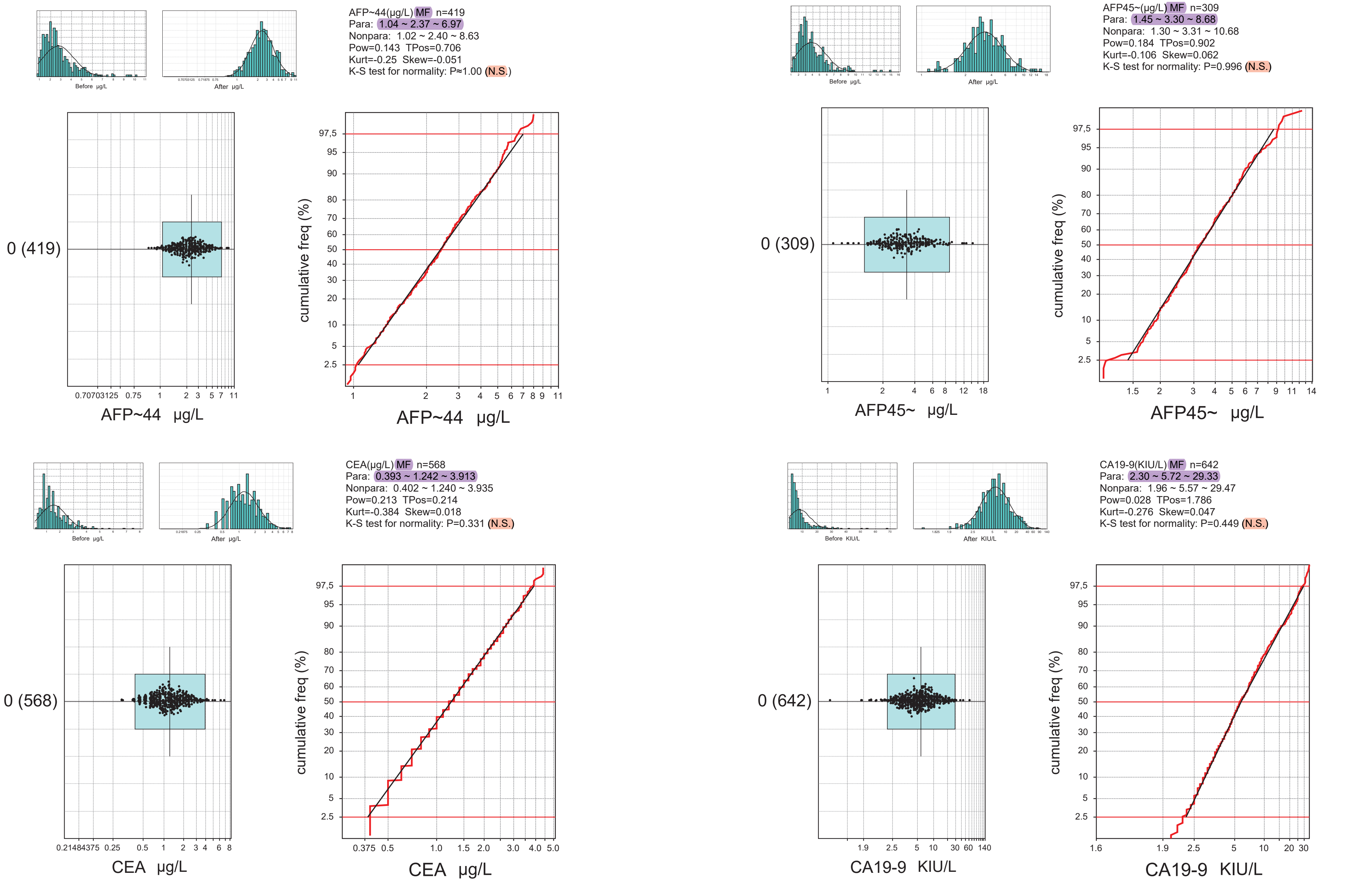

RIs were derived by both parametric and nonparametric methods. The accuracy of Gaussian transformation by Box-Cox formula can be assessed from theoretical Gaussian curves in two histograms shown on left top (before and after the transformation). The results of by Kolmogorov-Smirmov (K-S) test for normality of distribution were shown on right upper panel. The accuracy of the transformation can be also seen from the linearity in the probability paper plot on the right. The limits of the RI by nonparametric method corresponds to the points where red zigzag line intersect with horizontal 2.5 and 97.5 % red lines of cumulative frequencies.

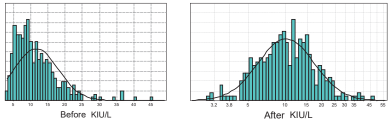

CA125(KIU/L) M n=341
Para: 3.94 ~ 10.05 ~ 27.42
Nonpara: 3.83 ~ 10.00 ~ 28.17
Pow=0.253 TPos=2.582
Kurt=-0.221 Skew=0.014
K-S test for normality: P≈1.00 (N.S.)

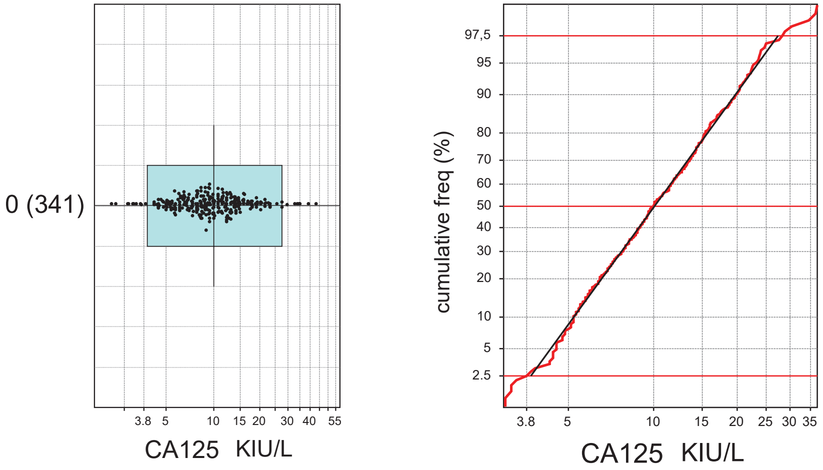

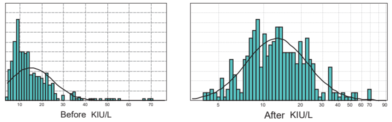

CA125(KIU/L) F n=391
Para: 4.81 ~ 12.34 ~ 38.78
Nonpara: 4.63 ~ 12.17 ~ 43.74
Pow=0.176 TPos=3.239
Kurt=-0.136 Skew=0.12
K-S test for normality: P=0.913 (N.S.)

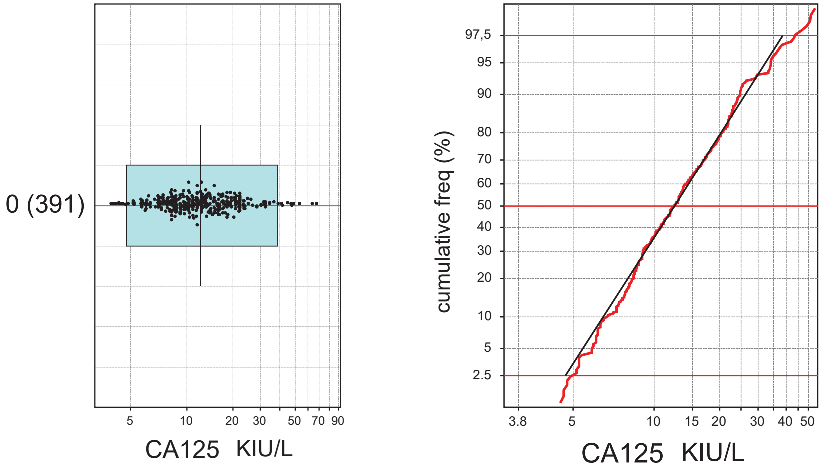

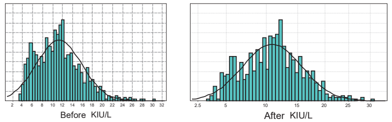

CA15-3(KIU/L) MF n=728
Para: 3.84 ~ 10.85 ~ 21.25
Nonpara: 4.52 ~ 11.00 ~ 24.21
Pow=0.537 TPos=0.177
Kurt=-0.537 Skew=0.041
K-S test for normality: P=0.277 (N.S.)

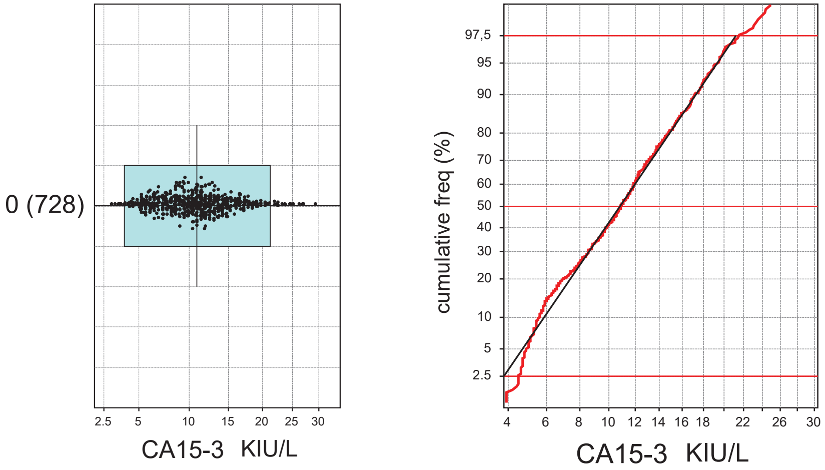

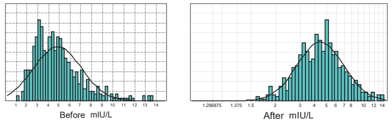

Insulin(mIU/L) MF n=504
Para: 1.95 ~ 4.39 ~ 10.49
Nonpara: 1.86 ~ 4.43 ~ 10.96
Pow=0.278 TPos=1.29
Kurt=-0.196 Skew=-0.072
K-S test for normality: P≈1.00 (N.S.)

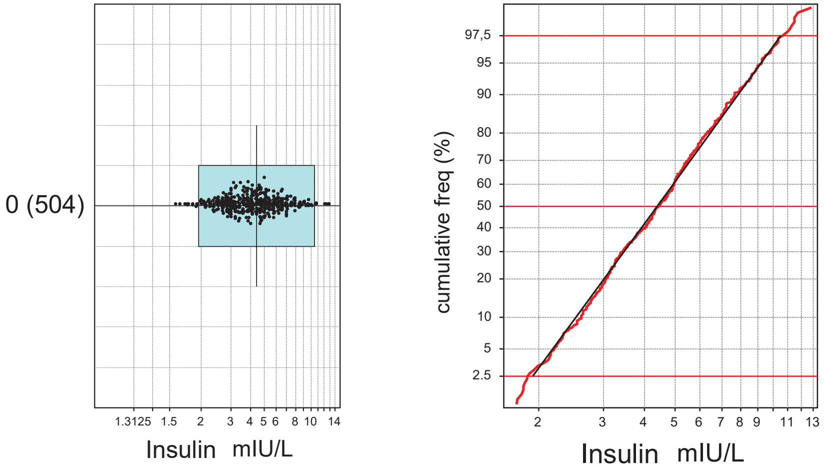

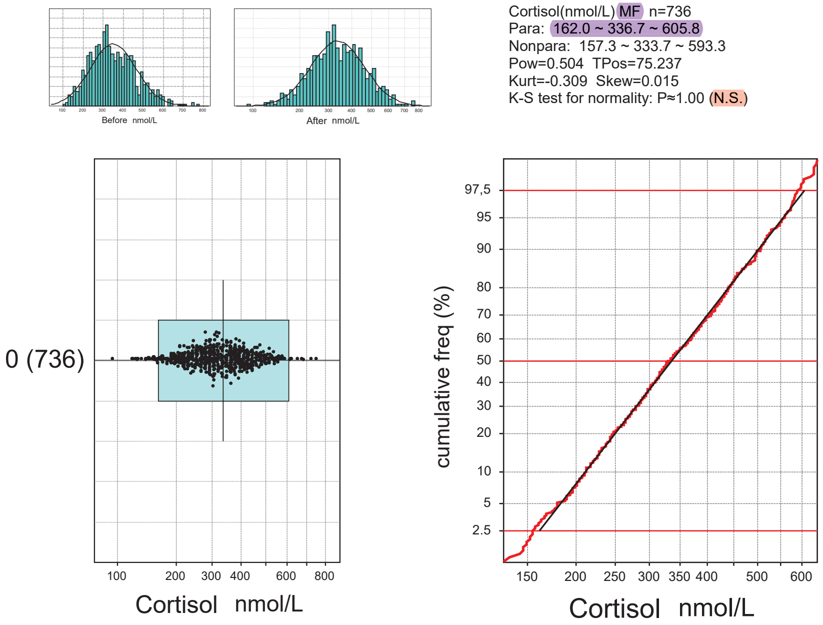
Cortisol(nmol/L) MF n=736
Para: 162.0 ~ 336.7 ~ 605.8
Nonpara: 157.3 ~ 333.7 ~ 593.3
Pow=0.504 TPos=75.237
Kurt=-0.309 Skew=0.015
K-S test for normality: P≈1.00 (N.S.)
Before nmol/L
After nmol/L
0 (736)
Cortisol nmol/L
cumulative freq (%)
Cortisol nmol/L

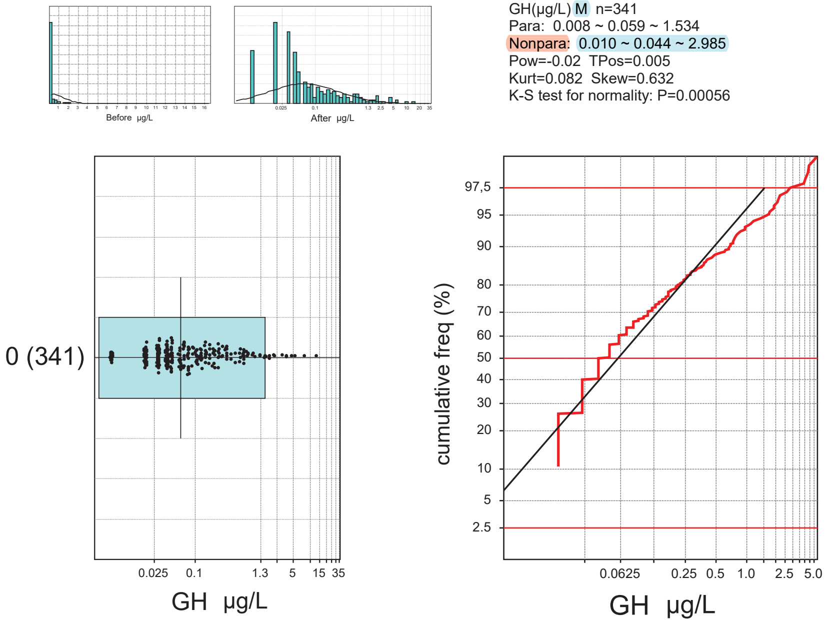
GH(μg/L) M n=341
Para: 0.008 ~ 0.059 ~ 1.534
Nonpara: 0.010 ~ 0.044 ~ 2.985
Pow=-0.02 TPos=0.005
Kurt=0.082 Skew=0.632
K-S test for normality: P=0.00056
Before μg/L
After μg/L
0 (341)
GH μg/L
cumulative freq (%)
GH μg/L

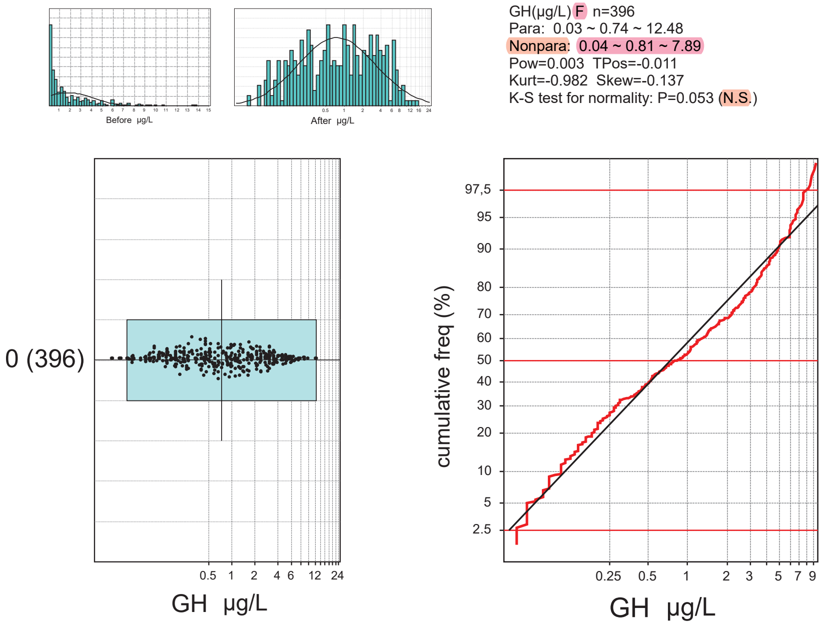
GH(μg/L) F n=396
Para: 0.03 ~ 0.74 ~ 12.48
Nonpara: 0.04 ~ 0.81 ~ 7.89
Pow=0.003 TPos=-0.011
Kurt=-0.982 Skew=-0.137
K-S test for normality: P=0.053 (N.S.)
Before μg/L
After μg/L
0 (396)
GH μg/L
cumulative freq (%)
GH μg/L

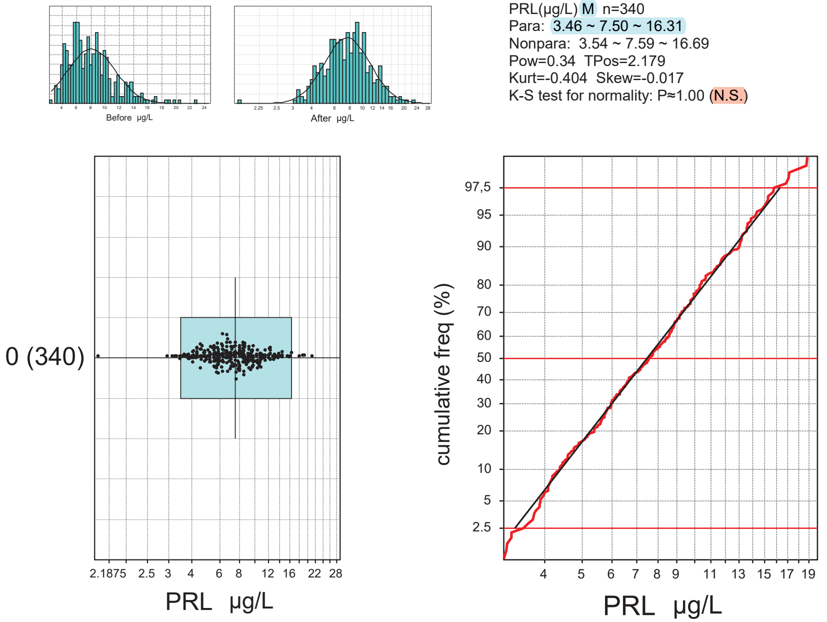
PRL(μg/L) M n=340
Para: 3.46 ~ 7.50 ~ 16.31
Nonpara: 3.54 ~ 7.59 ~ 16.69
Pow=0.34 TPos=2.179
Kurt=-0.404 Skew=-0.017
K-S test for normality: P≈1.00 (N.S.)
Before μg/L
After μg/L
0 (340)
PRL μg/L
cumulative freq (%)
PRL μg/L

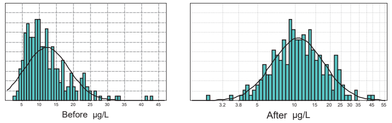

PRL(μg/L) F n=241
Para: 4.32 ~ 10.77 ~ 29.98
Nonpara: 4.31 ~ 10.66 ~ 33.52
Pow=0.211 TPos=2.603
Kurt=-0.29 Skew=0.045
K-S test for normality: P≈1.00 (N.S.)

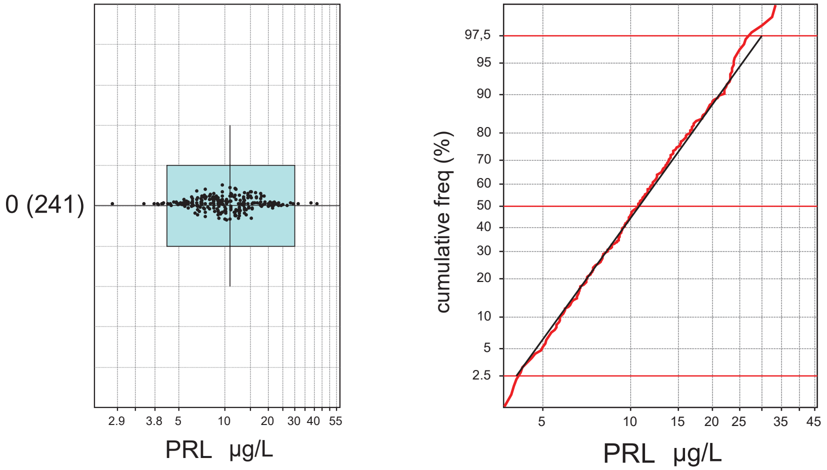

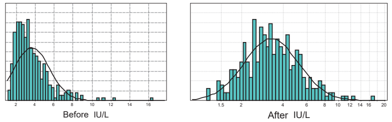

LH(IU/L) M n=332
Para: 1.39 ~ 3.17 ~ 8.12
Nonpara: 1.28 ~ 3.16 ~ 8.79
Pow=0.207 TPos=0.864
Kurt=-0.224 Skew=0.021
K-S test for normality: P≈1.00 (N.S.)

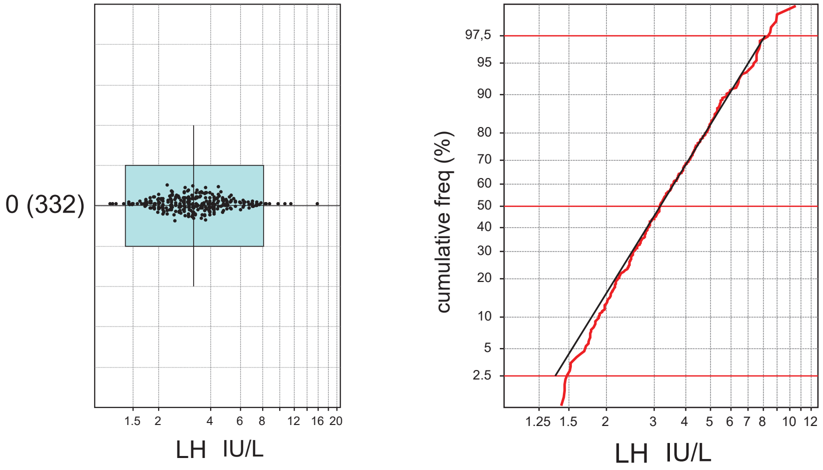

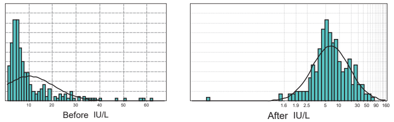

LH(IU/L) F n=242
Para: 2.02 ~ 6.70 ~ 42.48
Nonpara: 1.79 ~ 6.29 ~ 42.15
Pow=0.003 TPos=1.318
Kurt=-0.348 Skew=0.21
K-S test for normality: P=0.118 (N.S.)

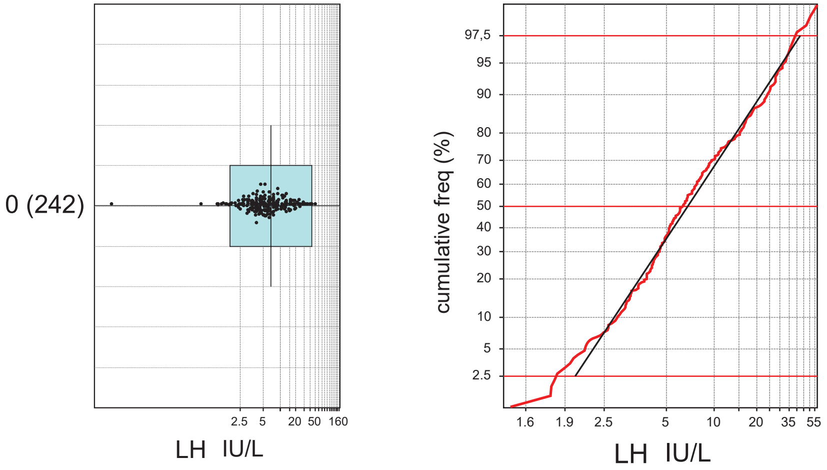

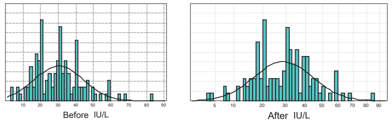

LH-MP(IU/L) F n=118
Para: 8.43 ~ 28.40 ~ 61.13
Nonpara: 7.41 ~ 29.40 ~ 62.91
Pow=0.514 TPos=-1.369
Kurt=-0.199 Skew=-0.104
K-S test for normality: P=0.804 (N.S.)

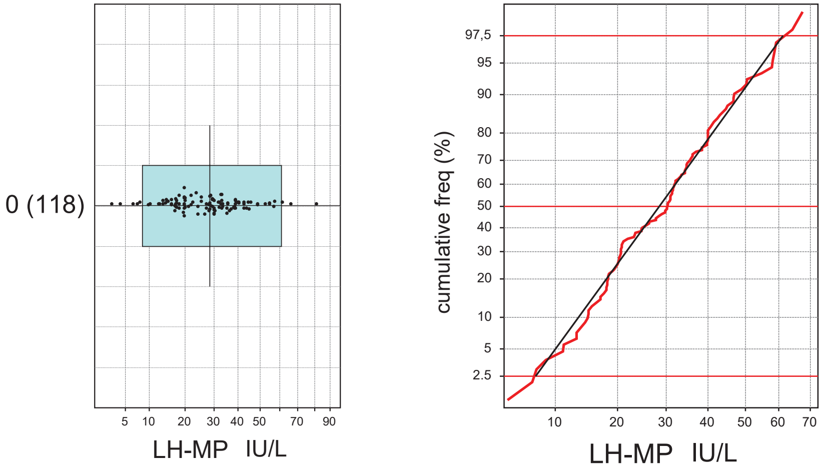

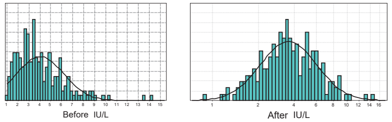

FSH~44(IU/L) M n=203
Para: 1.28 ~ 3.52 ~ 9.51
Nonpara: 1.26 ~ 3.55 ~ 9.64
Pow=0.315 TPos=0.853
Kurt=-0.332 Skew=-0.049
K-S test for normality: P≈1.00 (N.S.)

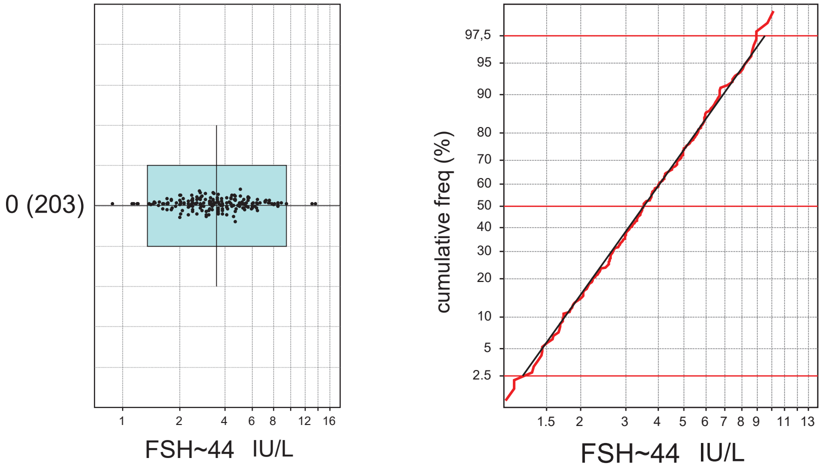

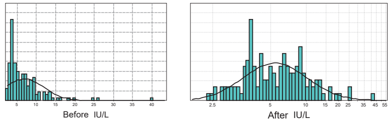

FSH45~(IU/L) M n=134
Para: 2.43 ~ 5.20 ~ 20.16
Nonpara: 2.32 ~ 5.18 ~ 20.14
Pow=0.076 TPos=1.941
Kurt=-0.343 Skew=0.027
K-S test for normality: P=0.655 (N.S.)

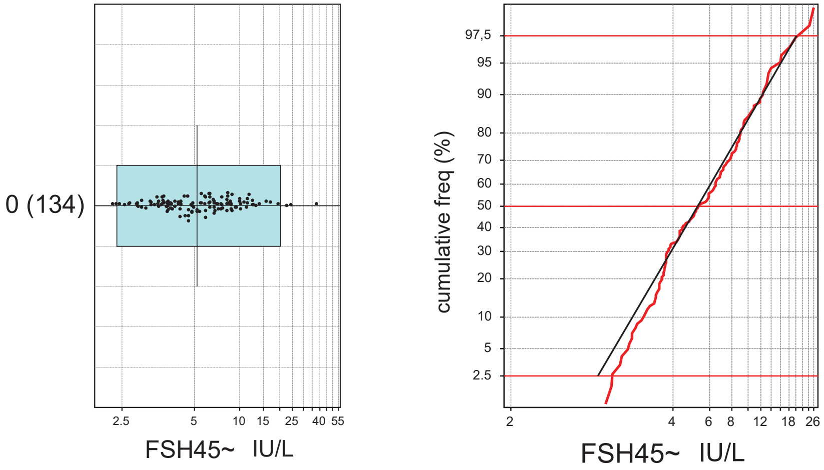

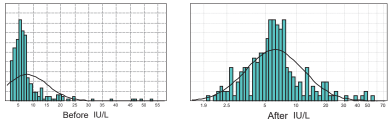

FSH(IU/L) F n=234
Para: 2.22 ~ 6.13 ~ 27.28
Nonpara: 2.22 ~ 6.11 ~ 63.44
Pow=0.107 TPos=1.403
Kurt=-0.058 Skew=-0.113
K-S test for normality: P=0.01270

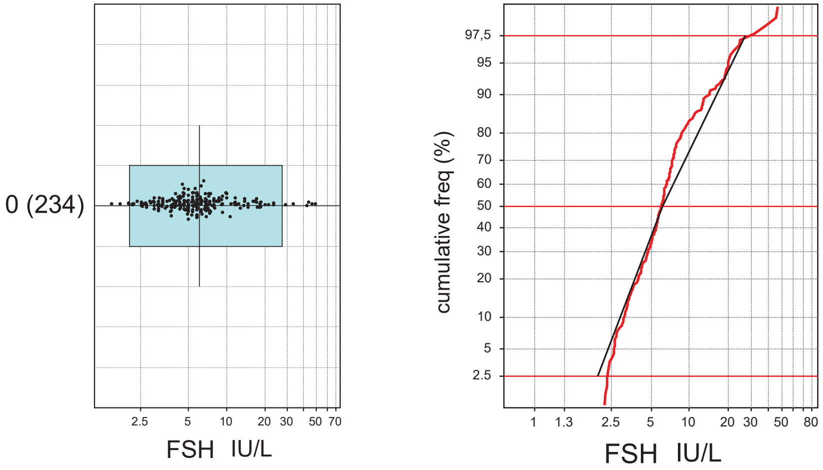

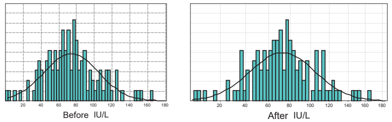

FSH-MP(IU/L) F n=118
Para: 21.1 ~ 72.7 ~ 137.5
Nonpara: 17.0 ~ 72.3 ~ 141.4
Pow=0.703 TPos=-18.274
Kurt=-0.217 Skew=0.058
K-S test for normality: P≈1.00 (N.S.)

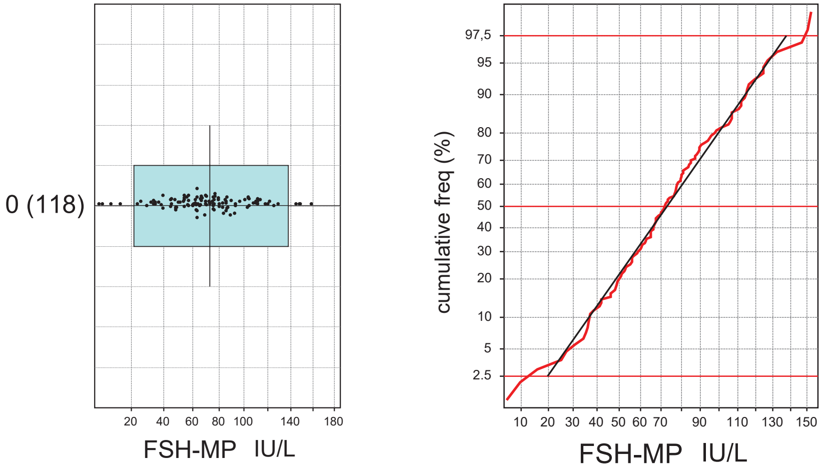

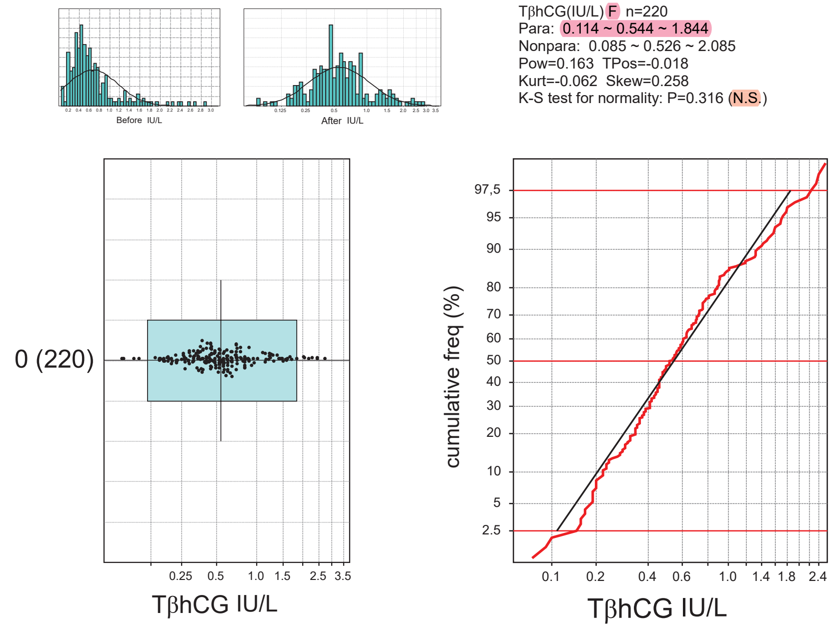
TβhCG(IU/L) F n=220
Para: 0.114 ~ 0.544 ~ 1.844
Nonpara: 0.085 ~ 0.526 ~ 2.085
Pow=0.163 TPos=-0.018
Kurt=-0.062 Skew=0.258
K-S test for normality: P=0.316 (N.S.)
Before IU/L
After IU/L
0 (220)
TβhCG IU/L
cumulative freq (%)
TβhCG IU/L

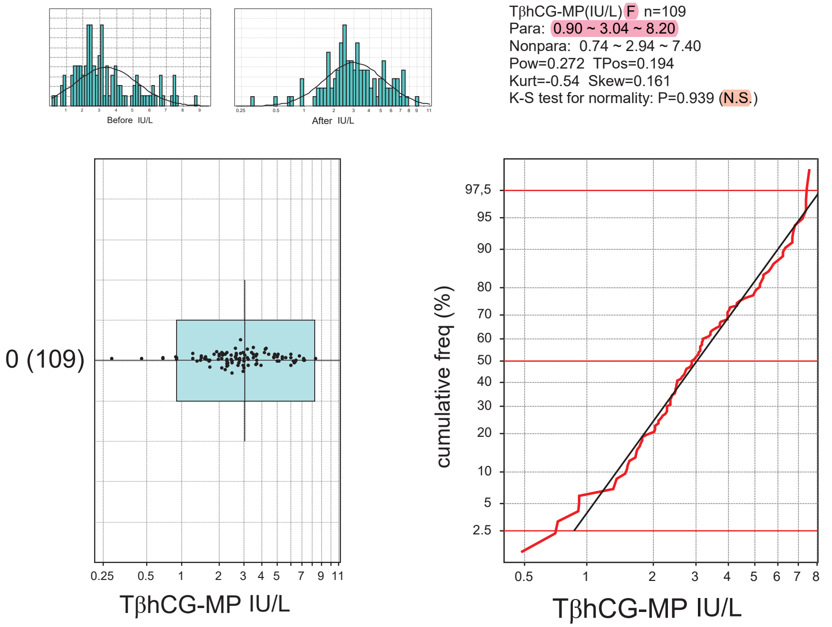
TβhCG-MP(IU/L) F n=109
Para: 0.90 ~ 3.04 ~ 8.20
Nonpara: 0.74 ~ 2.94 ~ 7.40
Pow=0.272 TPos=0.194
Kurt=-0.54 Skew=0.161
K-S test for normality: P=0.939 (N.S.)
Before IU/L
After IU/L
0 (109)
TβhCG-MP IU/L
cumulative freq (%)
TβhCG-MP IU/L

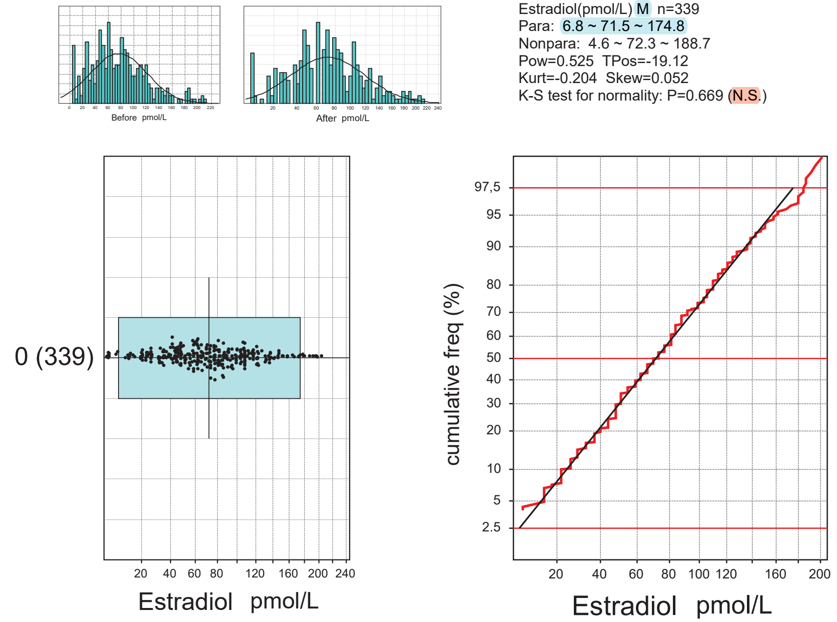
Estradiol(pmol/L) M n=339
Para: 6.8 ~ 71.5 ~ 174.8
Nonpara: 4.6 ~ 72.3 ~ 188.7
Pow=0.525 TPos=-19.12
Kurt=-0.204 Skew=0.052
K-S test for normality: P=0.669 (N.S.)
Before pmol/L
After pmol/L
0 (339)
Estradiol pmol/L
cumulative freq (%)
Estradiol pmol/L

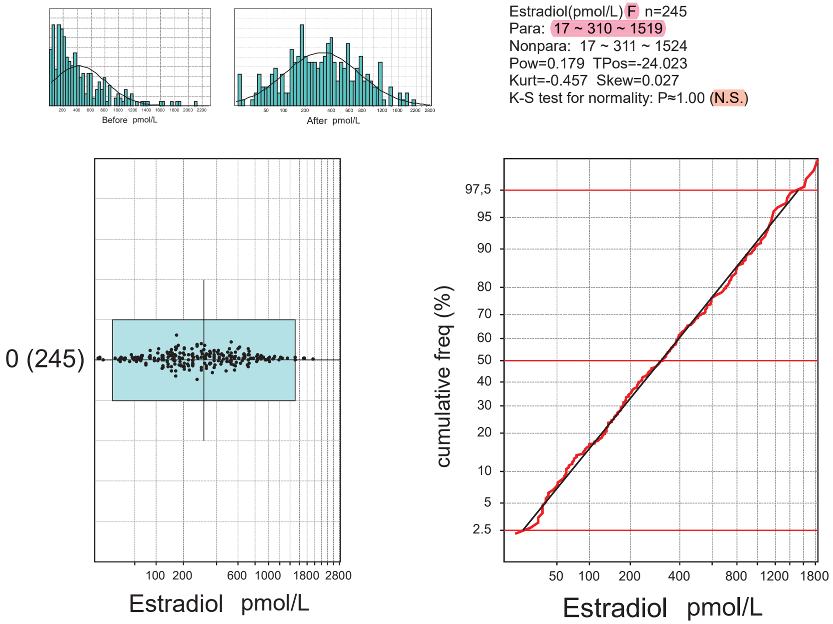
Estradiol(pmol/L) F n=245
Para: 17 ~ 310 ~ 1519
Nonpara: 17 ~ 311 ~ 1524
Pow=0.179 TPos=-24.023
Kurt=-0.457 Skew=0.027
K-S test for normality: P≈1.00 (N.S.)
Before pmol/L
After pmol/L
0 (245)
Estradiol pmol/L
cumulative freq (%)
Estradiol pmol/L

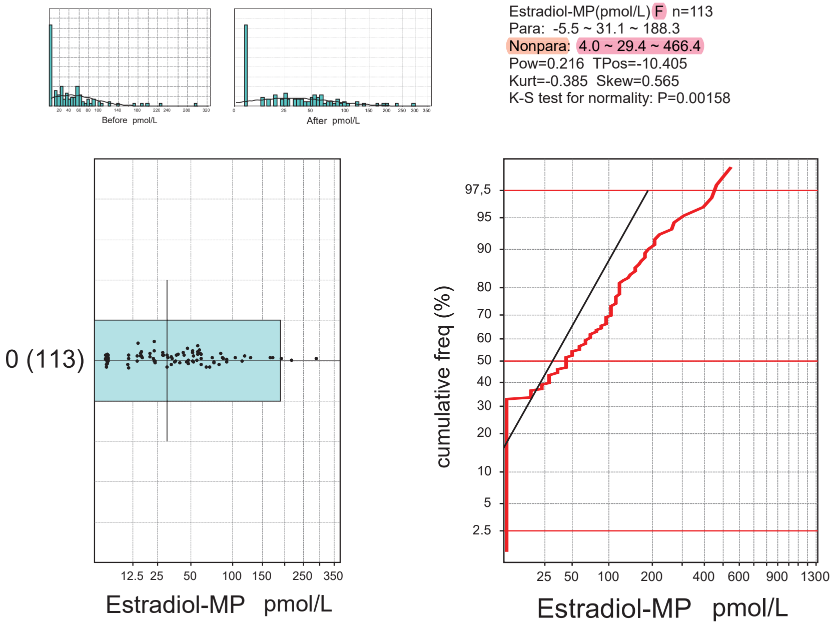
Estradiol-MP(pmol/L) F n=113
Para: -5.5 ~ 31.1 ~ 188.3
Nonpara: 4.0 ~ 29.4 ~ 466.4
Pow=0.216 TPos=-10.405
Kurt=-0.385 Skew=0.565
K-S test for normality: P=0.00158
Before pmol/L
After pmol/L
0 (113)
Estradiol-MP pmol/L
cumulative freq (%)
Estradiol-MP pmol/L

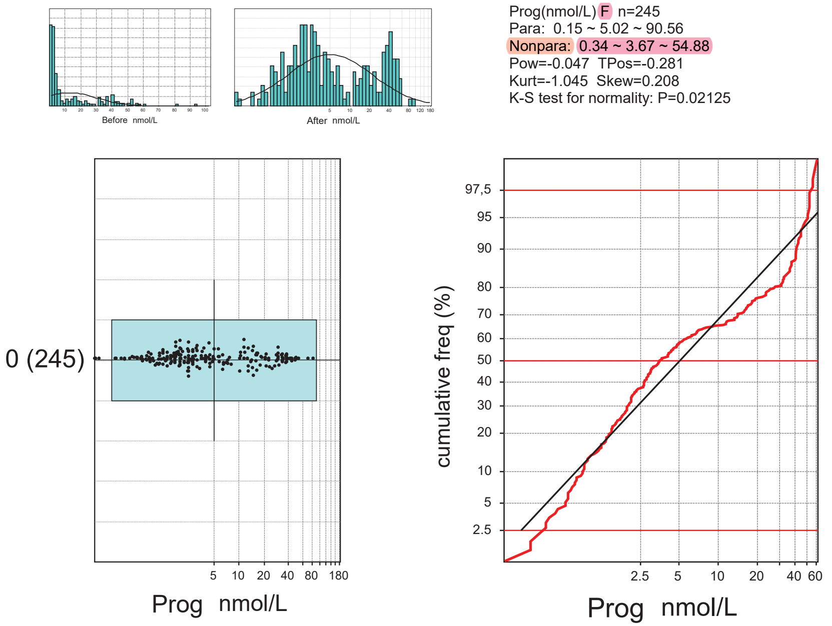
Prog(nmol/L) F n=245
Para: 0.15 ~ 5.02 ~ 90.56
Nonpara: 0.34 ~ 3.67 ~ 54.88
Pow=-0.047 TPos=-0.281
Kurt=-1.045 Skew=0.208
K-S test for normality: P=0.02125
Before nmol/L
After nmol/L
0 (245)
Prog nmol/L
cumulative freq (%)
Prog nmol/L

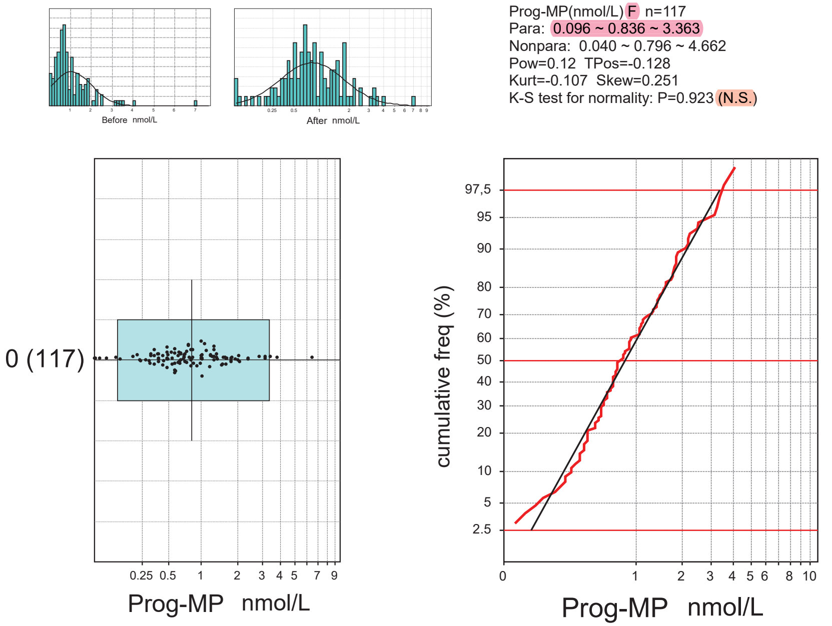
Prog-MP(nmol/L) F n=117
Para: 0.096 ~ 0.836 ~ 3.363
Nonpara: 0.040 ~ 0.796 ~ 4.662
Pow=0.12 TPos=-0.128
Kurt=-0.107 Skew=0.251
K-S test for normality: P=0.923 (N.S.)
Before nmol/L
After nmol/L
0 (117)
Prog-MP nmol/L
cumulative freq (%)
Prog-MP nmol/L

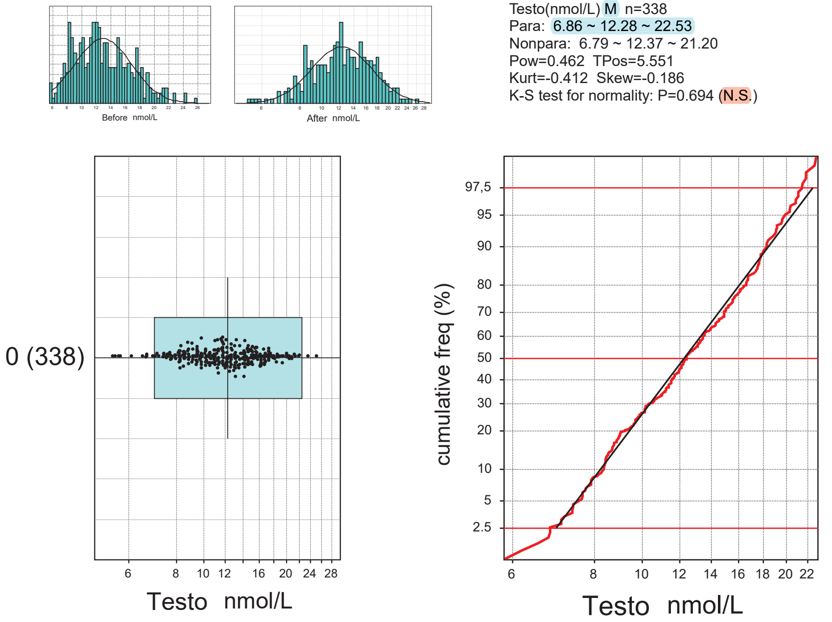
Testo(nmol/L) M n=338
Para: 6.86 ~ 12.28 ~ 22.53
Nonpara: 6.79 ~ 12.37 ~ 21.20
Pow=0.462 TPos=5.551
Kurt=-0.412 Skew=-0.186
K-S test for normality: P=0.694 (N.S.)
Before nmol/L
After nmol/L
0 (338)
Testo nmol/L
cumulative freq (%)
Testo nmol/L

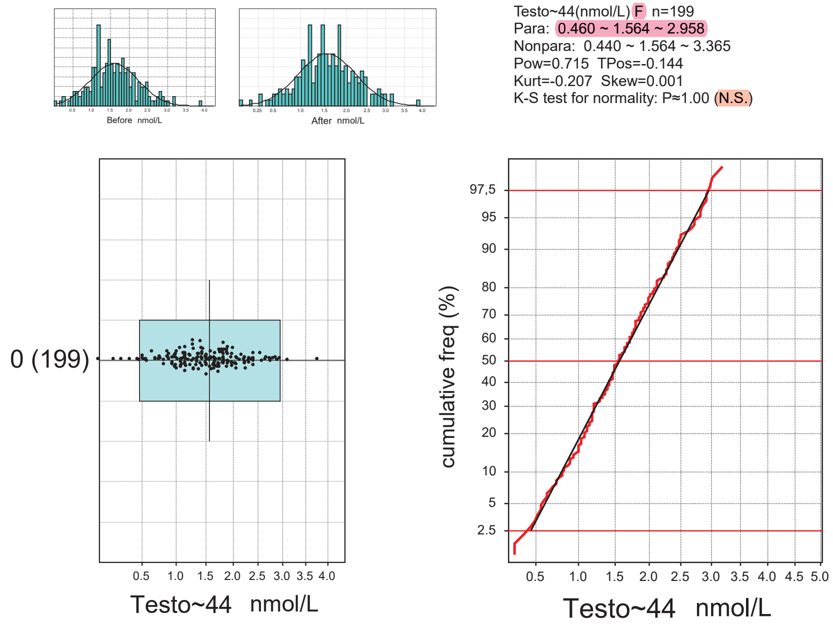
Testo~44(nmol/L) F n=199
Para: 0.460 ~ 1.564 ~ 2.958
Nonpara: 0.440 ~ 1.564 ~ 3.365
Pow=0.715 TPos=-0.144
Kurt=-0.207 Skew=0.001
K-S test for normality: P≈1.00 (N.S.)
Before nmol/L
After nmol/L
0 (199)
Testo~44 nmol/L
cumulative freq (%)
Testo~44 nmol/L

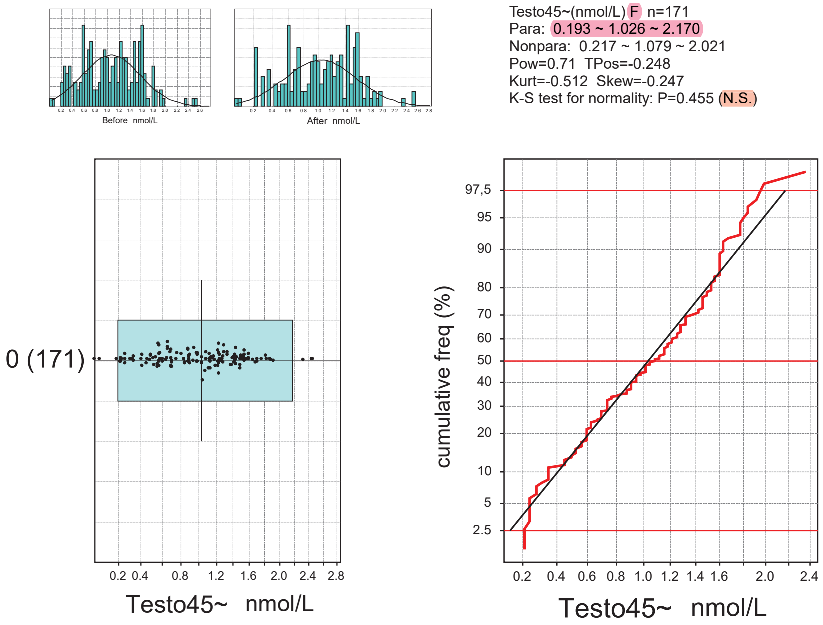
Testo45~(nmol/L) F n=171
Para: 0.193 ~ 1.026 ~ 2.170
Nonpara: 0.217 ~ 1.079 ~ 2.021
Pow=0.71 TPos=-0.248
Kurt=-0.512 Skew=-0.247
K-S test for normality: P=0.455 (N.S.)
Before nmol/L
After nmol/L
0 (171)
Testo45~ nmol/L
cumulative freq (%)
Testo45~ nmol/L

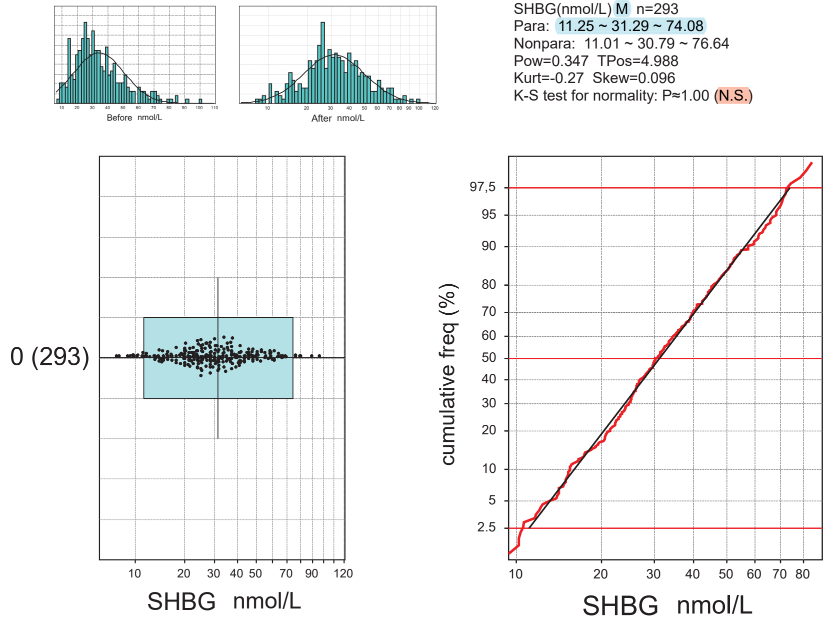
SHBG(nmol/L) M n=293
Para: 11.25 ~ 31.29 ~ 74.08
Nonpara: 11.01 ~ 30.79 ~ 76.64
Pow=0.347 TPos=4.988
Kurt=-0.27 Skew=0.096
K-S test for normality: P≈1.00 (N.S.)
Before nmol/L
After nmol/L
0 (293)
SHBG nmol/L
cumulative freq (%)
SHBG nmol/L

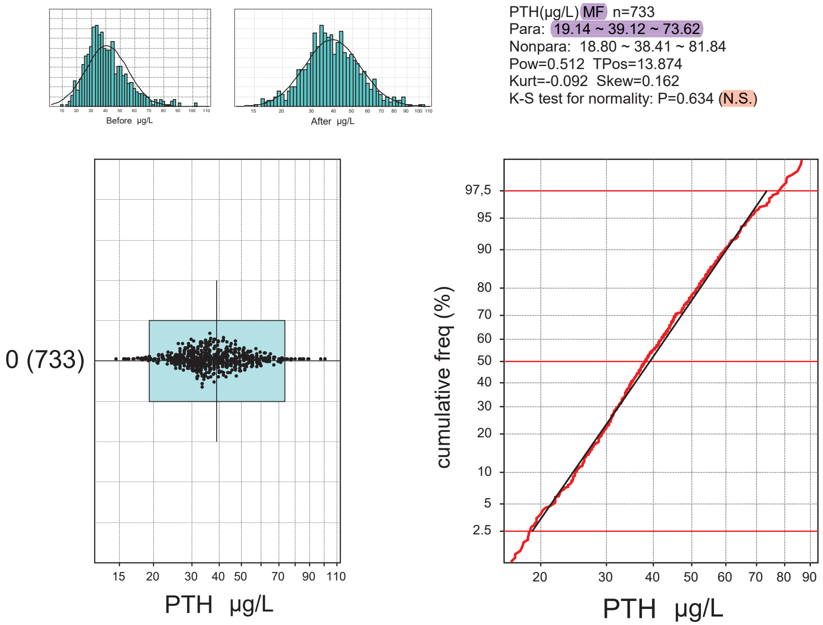
PTH(μg/L) MF n=733
Para: 19.14 ~ 39.12 ~ 73.62
Nonpara: 18.80 ~ 38.41 ~ 81.84
Pow=0.512 TPos=13.874
Kurt=-0.092 Skew=0.162
K-S test for normality: P=0.634 (N.S.)
Before μg/L
After μg/L
0 (733)
PTH μg/L
cumulative freq (%)
PTH μg/L

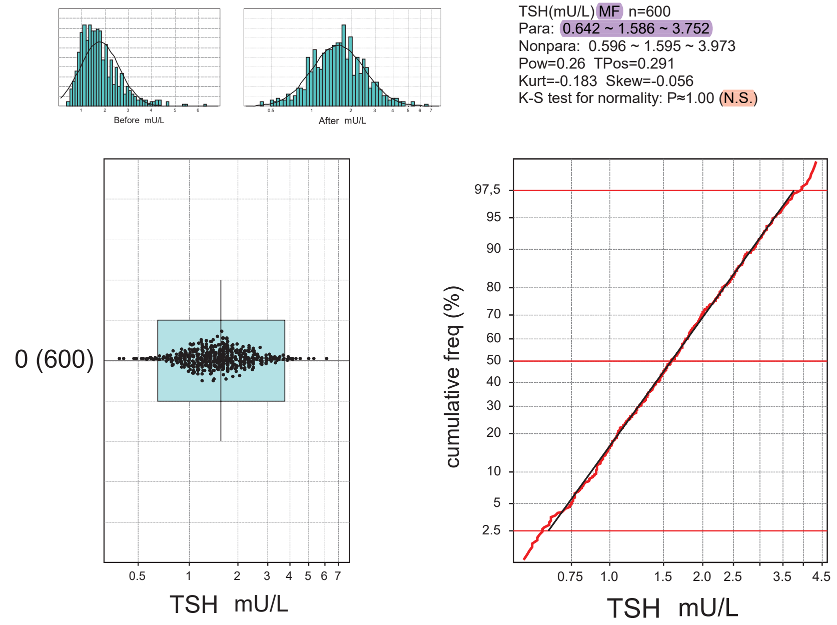
TSH(mU/L) MF n=600
Para: 0.642 ~ 1.586 ~ 3.752
Nonpara: 0.596 ~ 1.595 ~ 3.973
Pow=0.26 TPos=0.291
Kurt=-0.183 Skew=-0.056
K-S test for normality: P≈1.00 (N.S.)
Before mU/L
After mU/L
0 (600)
TSH mU/L
cumulative freq (%)
TSH mU/L

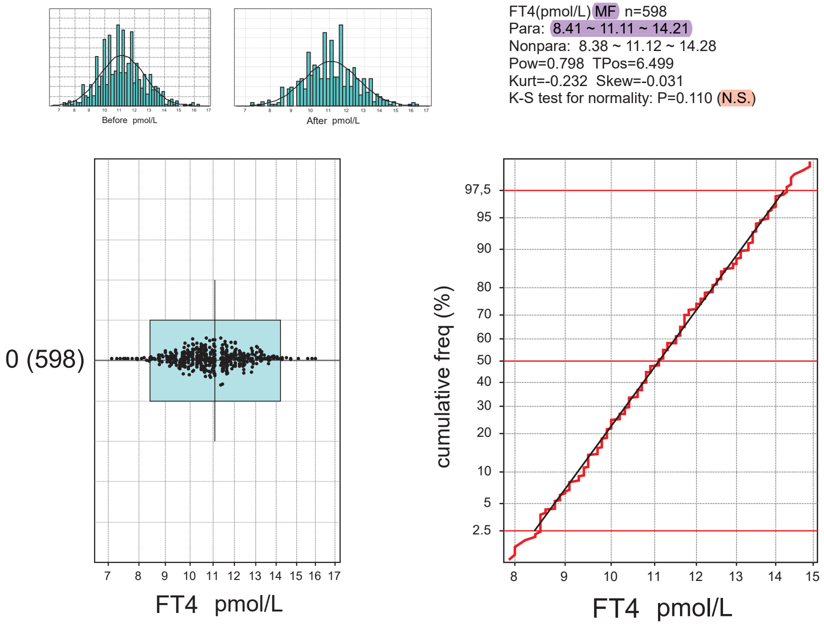
FT4(pmol/L) MF n=598
Para: 8.41 ~ 11.11 ~ 14.21
Nonpara: 8.38 ~ 11.12 ~ 14.28
Pow=0.798 TPos=6.499
Kurt=-0.232 Skew=-0.031
K-S test for normality: P=0.110 (N.S.)
Before pmol/L
After pmol/L
0 (598)
FT4 pmol/L
cumulative freq (%)
FT4 pmol/L

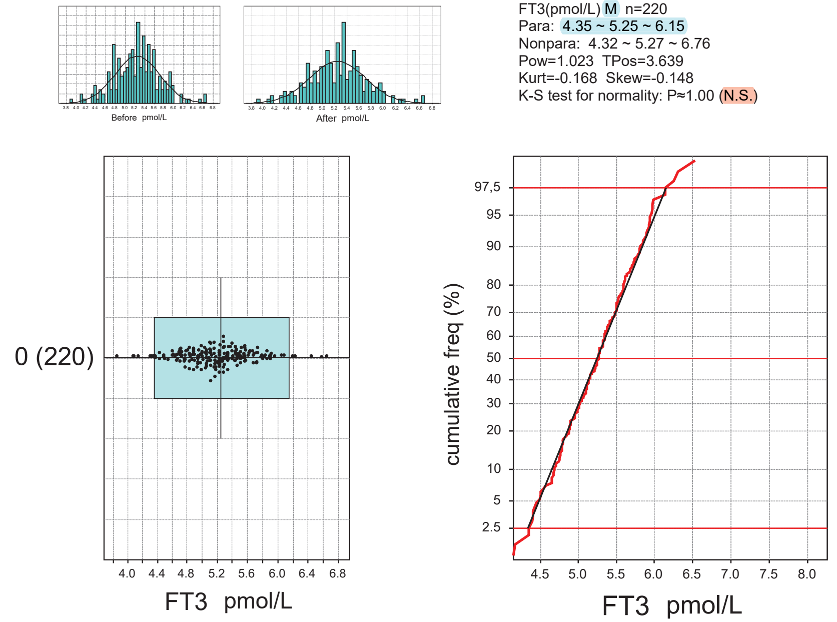
FT3(pmol/L) M n=220
Para: 4.35 ~ 5.25 ~ 6.15
Nonpara: 4.32 ~ 5.27 ~ 6.76
Pow=1.023 TPos=3.639
Kurt=-0.168 Skew=-0.148
K-S test for normality: P≈1.00 (N.S.)
Before pmol/L
After pmol/L
0 (220)
FT3 pmol/L
cumulative freq (%)
FT3 pmol/L

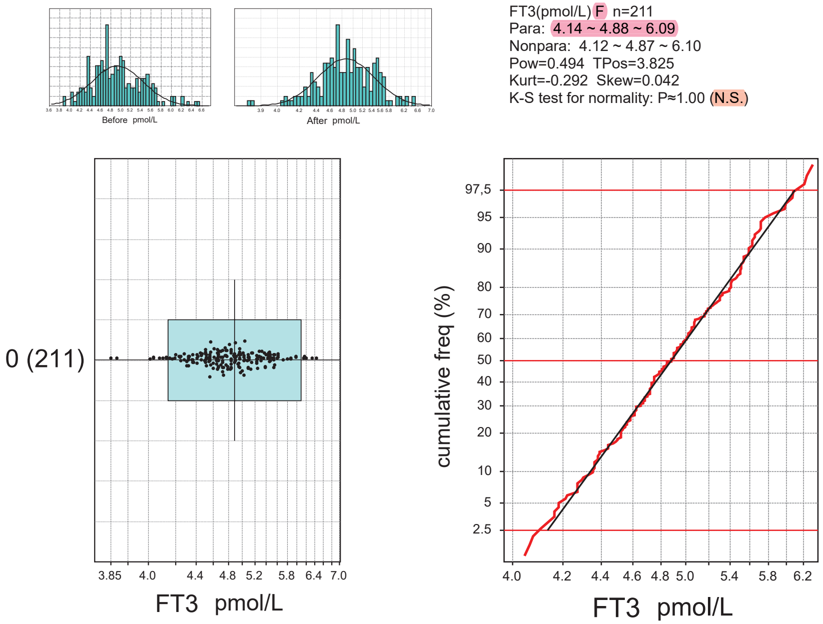
FT3(pmol/L) F n=211
Para: 4.14 ~ 4.88 ~ 6.09
Nonpara: 4.12 ~ 4.87 ~ 6.10
Pow=0.494 TPos=3.825
Kurt=-0.292 Skew=0.042
K-S test for normality: P≈1.00 (N.S.)
Before pmol/L
After pmol/L
0 (211)
FT3 pmol/L
cumulative freq (%)
FT3 pmol/L

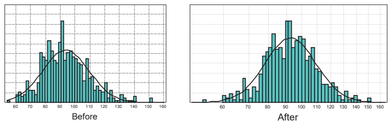

TT4 MF n=567
Para: 66.6 ~ 92.7 ~ 127.4
Nonpara: 65.4 ~ 92.2 ~ 130.3
Pow=0.641 TPos=52.51
Kurt=-0.093 Skew=0.011
K-S test for normality: P=0.709 (N.S.)

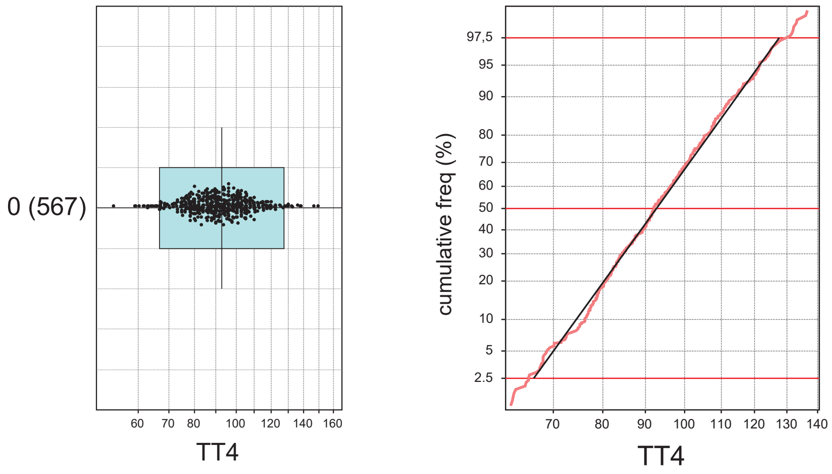

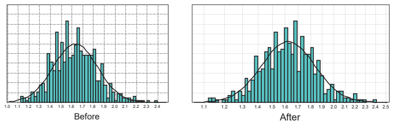

TT3 MF n=561
Para: 1.266 ~ 1.619 ~ 2.058
Nonpara: 1.248 ~ 1.620 ~ 2.108
Pow=0.703 TPos=1.028
Kurt=-0.119 Skew=-0.016
K-S test for normality: P≈1.00 (N.S.)

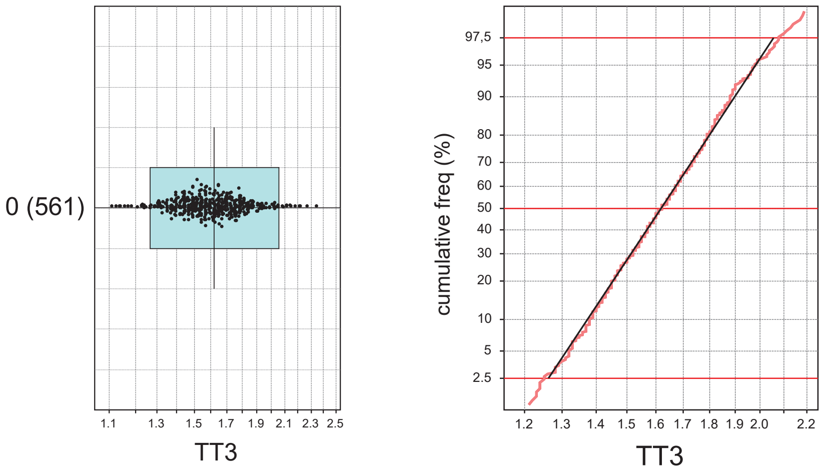

Supplement: S2 Fig — RIs were derived by both parametric and nonparametric methods. The accuracy of Gaussian transformation by Box-Cox formula can be assessed from theoretical Gaussian curves in two histograms shown on left top (before and after the transformation). The results of by Kolmogorov-Smirmov (K-S) test for normality of distribution were shown on right upper panel. The accuracy of the transformation can be also seen from the linearity in the probability paper plot on the right. The limits of the RI by nonparametric method corresponds to the points where red zigzag line intersect with horizontal 2.5 and 97.5% red lines of cumulative frequencies. (PDF) [file pone.0234284.s003.pdf]
