# Supplementary material for: Sources of variation and establishment of Russian reference intervals for major hormones and tumor markers
Source: PLoS One. 2021 Jan 7;16(1):e0234284. doi: 10.1371/journal.pone.0234284 (PMC7790266; doi:10.1371/journal.pone.0234284)
Supplement: S3 Fig — Aliquots of volunteers’ sera stored at −80C° were tested in 2018 by use of new reagents for TSH and TβhCG after confirmation of the stability of the analytes. Recalibration of values by the old reagent was performed using the major-axis linear regression between new and old values after logarithmic and square-root transformation for TSH and TβhCG, respectively. (PDF) [file pone.0234284.s004.pdf]

**S3 Fig. Comparison of test results for TSH and TβhCG before and after reagent changes**

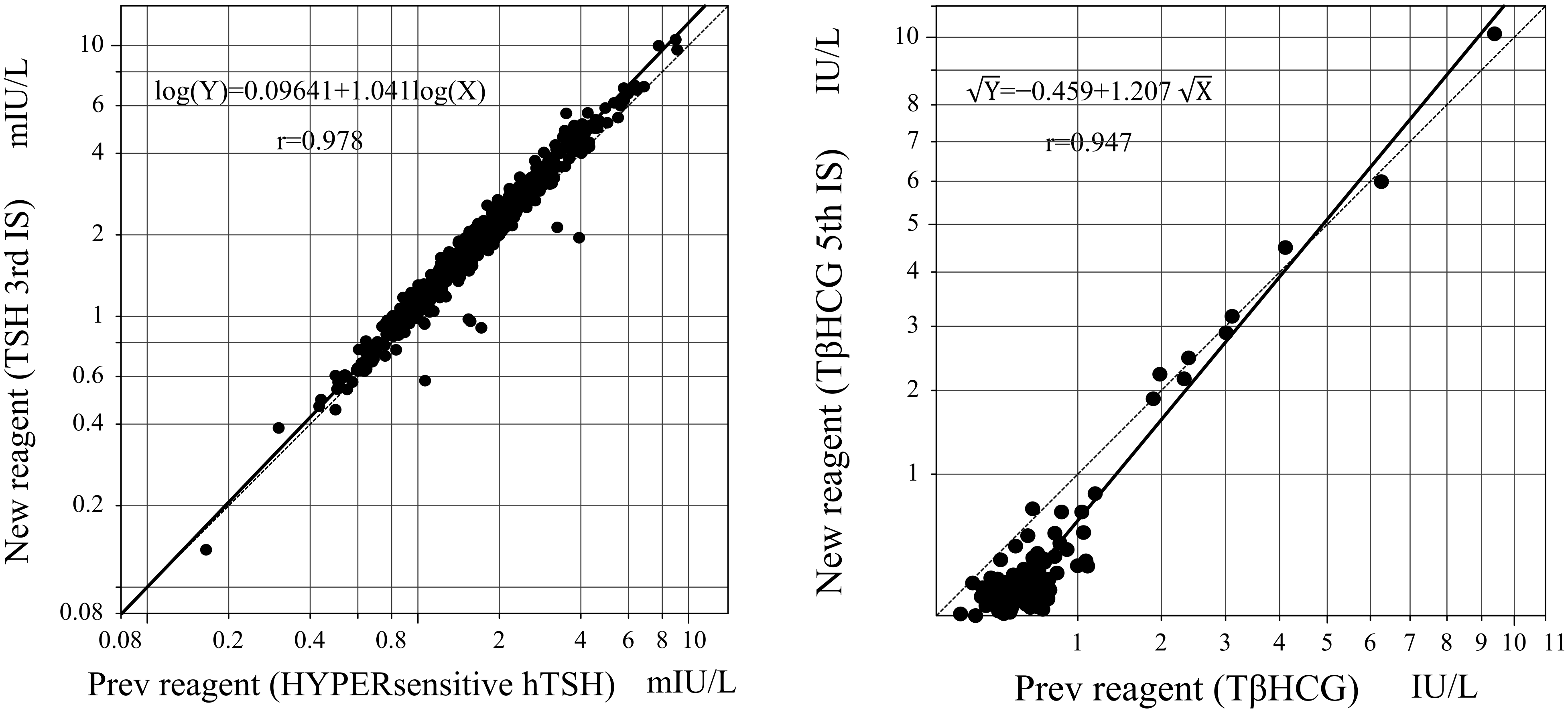


| | pre-MP | post-MP |
|---|---|---|
| New reagent RI: | 0.11~1.84, | 0.90~8.20 IU/L |
| Prev reagent RI: | 0.44~2.27, | 1.36~7.58 IU/L |
