# Supplementary material for: Sources of variation and establishment of Russian reference intervals for major hormones and tumor markers
Source: PLoS One. 2021 Jan 7;16(1):e0234284. doi: 10.1371/journal.pone.0234284 (PMC7790266; doi:10.1371/journal.pone.0234284)
Supplement: S1 Table — (PDF) [file pone.0234284.s005.pdf]

**S1 Table Demographic profile of volunteers**

| | | | | | |
|---|---|---|---|---|---|
| **Saint-Petersburg** | **Sex** | **Males** | | **Females** | |
| | | 234 (46%) | | 272 (54%) | |
| | **Age** | **< 45** | **≥ 45** | **< 45** | **≥ 45** |
| | | 146 (62%) | 88 (38%) | 155 (57%) | 117 (43%) |
| | **BMI** | **< 28** | **≥ 28** | **< 28** | **≥ 28** |
| | | 160 (68%) | 74 (32%) | 188 (69%) | 84 (31%) |
| | **Total** | **506 (67%)** | | | |
| **Moscow** | **Sex** | **Males** | | **Females** | |
| | | 51 (43%) | | 66 (56%) | |
| | **Age** | **< 45** | **≥ 45** | **< 45** | **≥ 45** |
| | | 30 (59%) | 21 (41%) | 34 (51%) | 32 (49%) |
| | **BMI** | **< 28** | **≥ 28** | **< 28** | **≥ 28** |
| | | 28 (55%) | 23 (45%) | 44 (67%) | 22 (33%) |
| | **Total** | **117 (15%)** | | | |
| **Ekaterinburg** | **Sex** | **Males** | | **Females** | |
| | | 65 (48%) | | 70 (52%) | |
| | **Age** | **< 45** | **≥ 45** | **< 45** | **≥ 45** |
| | | 33 (51%) | 32 (49%) | 38 (54%) | 32 (46%) |
| | **BMI** | **< 28** | **≥ 28** | **< 28** | **≥ 28** |
| | | 45 (69%) | 20 (31%) | 54 (77%) | 16 (23%) |
| | **Total** | **135 (18%)** | | | |
| **Total** | **Sex** | **Males** | | **Females** | |
| | | 350 (46%) | | 408 (54%) | |
| | **Age** | **< 45** | | **≥ 45** | |
| | | 209 (60%) | 141 (40%) | 227 (56%) | 181 (44%) |
| | **BMI** | **< 28** | | **≥ 28** | |
| | | 233 (67%) | 117 (33%) | 286 (70%) | 122 (30%) |
| | **Total** | **758** | | | |
