# Supplementary material for: Sources of variation and establishment of Russian reference intervals for major hormones and tumor markers
Source: PLoS One. 2021 Jan 7;16(1):e0234284. doi: 10.1371/journal.pone.0234284 (PMC7790266; doi:10.1371/journal.pone.0234284)
Supplement: S2 Table — (PDF) [file pone.0234284.s006.pdf]

S2 Table Characteristics of assays for analytes examined in this study

Analyte	Abbr	Units	Assay Principle	Tracebility	Between-day CV	CVi/2 (EFLM)	CV Limits (EFLM)
**Anti-thyroid peroxidase antibody**	TPOAb	kIU/L	ICLA*	International standard WHO 66/387	3.2	na	na
**Anti-thyroglobulin antibody**	TgAb	kIU/L	ICLA	WHO 65/93 International Standard	na	na	na
**Alpha-fetoprotein**	AFP	µg/L	ICLA	WHO 1 st International Standard 72/225	8.2	13.4	23.8 - 30.3
**Carcinoembryonic antigen**	CEA	µg/L	ICLA	Manufacturer's working calibrators	7.5	9.0	12.6 - 30.9
**CA 19-9**	CA 19-9	kIU/L	ICLA	Manufacturer's working calibrators	3.6	11.3	15.8 - 27.2
**CA 125**	CA 125	kIU/L	ICLA	Manufacturer's working calibrators	4.4	6.7	9.1 - 23.3
**CA 15-3**	CA 15-3	kIU/L	ICLA	Manufacturer's working calibrators	2.7	na	na
**Insulin**	Insulin	mIU/L	ICLA	WHO 1 st International Reference Preparation 66/304	6.8	14.3	21.1 - 37.1
**Cortisol**	Cortisol	nmol/L	ICLA	USP reference material	4.2	11.6	21.7 - 26.6
**Growth hormone**	GH	µg/L	ICLA	WHO second international standard (2nd IS), WHO 98/574	2.6	na	na
**Prolactin**	PRL	µg/L	ICLA	WHO 3rd International Standard for Prolactin (84/500)	2.4	10.0	17.1 - 23.6
**Luteinizing hormone**	LH	IU/L	ICLA	WHO 2 nd International Reference Preparation for hLH (80/552)	5.4	11.4	22 - 24
**Follicle stimulating hormone**	FSH	IU/L	ICLA	WHO 2nd International Reference Preparation for hFSH (78/549)	4.4	6.2	11 - 17.3
**Total beta human chorionic gonadotropin**	TβhCG	IU/L	ICLA	WHO 5 th International Standard for Chorionic Gonadotropin (NIBSC Code 07/364)	3.0	na	na
**Estradiol**	Estradiol	pmol/l	ICLA	Joint Committee for Traceability in Laboratory Medicine (JCTLM)-approved isotope dilution mass spectrometry (ID/GC/MS) reference method procedure (RMP)	2.5	7.5	13.3 - 16.5
**Progesterone**	Prog	nmol/l	ICLA	in Laboratory Medicine (JCTLM)-approved isotope dilution mass spectrometry (ID/GC/MS) reference method procedure	6.2	na	na
**Testosterone**	Testo	nmol/l	ICLA	USP reference material	2.0	10.7	10.9 - 15.1
**Sex hormone-binding protein**	SHBG		ICLA	WHO 95/560	4.6	4.9	4.3 - 14.0
**Parathyroid hormone**	PTH	µg/L	ICLA	Manufacturer's working calibrators	3.1	7.9	14.7 - 25.9
**Thyroid stimulating hormone**	TSH	mU/L	ICLA	3rd International standard 81/565 WHO	3.2	10.6	14.8 - 29.3
**Free thyroxine**	FT4	pmol/L	ICLA	Manufacturer's working calibrators	3.2	3.9	4.8 - 9.5
**Free triiodothyronine**	FT3	pmol/L	ICLA	Manufacturer's working calibrators	3.1	3.0	4.7 - 7.9
**Total thyroxine**	TT4	nmol/L	ICLA	USP Levothyroxine reference material	3.2	3.2	4.9 - 7.4
**Total triiodothyronine**	TT3	nmol/L	ICLA	USP reference material	4.6	4.7	6.9 - 10.4

*ICLA - immunochemiluminescent assay (Beckman Coulter)
