# Supplementary material for: Sources of variation and establishment of Russian reference intervals for major hormones and tumor markers
Source: PLoS One. 2021 Jan 7;16(1):e0234284. doi: 10.1371/journal.pone.0234284 (PMC7790266; doi:10.1371/journal.pone.0234284)
Supplement: S3 Table — (PDF) [file pone.0234284.s007.pdf]

S3 Table Comparison of assay characteristics for TSH and TβhCG before and after r eagent changes

Assay name	TSH		TbHCG	
	**Access HYPERsensitive hTSH**	**Access TSH (3rd IS)**	**Access Total ßhCG**	**Total βhCG (5th IS)**
**Reagent part number**	33820	B63284	33500	A85264
**Assay Format**	Two-site immunoenzymatic ("sandwich") assay	Two-site immunoenzymatic ("sandwich") assay	1-step (sandwich)	2-step sequential (sandwich)
**Tracebility**	WHO 2nd International Reference Preparation 80/558".	WHO 3rd International standard (IS) for human TSH (IRP 81/565)	WHO 3rd International standard (IS) WHO 75/537	WHO 5 th International Standard for Chorionic Gonadotropin (NIBSC Code 07/364)
**Reportable Range (Including Dilution, approximate)**	0.015 to 100.0 µIU/mL	0.005 to 50.0 µIU/mL	0,5–1000 mIU/mL	0.5 - 1.350 mIU/mL
**The dilution rate**	Up to 500 µIU/mL	Up to 500 µIU/mL	Up to ~ 200,000 mIU/mL	Up to ~ 270,000 mIU/mL
**Pack size**	100 tests/pack	200 tests/pack	100 tests/pack	100 tests/pack
