# Supplementary material for: Sources of variation and establishment of Russian reference intervals for major hormones and tumor markers
Source: PLoS One. 2021 Jan 7;16(1):e0234284. doi: 10.1371/journal.pone.0234284 (PMC7790266; doi:10.1371/journal.pone.0234284)
Supplement: S4 Table — (PDF) [file pone.0234284.s008.pdf]

S4 Table List of RIs derived in various conditions partitioned by sex and/or age (menopausal state) with/without secondary exclusion options.

	All=18–64					Males + Females					Males					Females				BR-Sex		BR-age MF (BRbmi MF)		BR-age M (BRbmi M)		BR-age F (BRbmi F)		Decision
Stat	Item	Units	Age	Other excl criteria		n	LL	Me	UL		n	LL	Me	UL		n	LL	Me	UL	LL	UL	LL	UL	LL	UL	LL	UL	
P	AFP	μg/L	All			660	1.1	2.8	7.5		312	1.3	2.9	7.0		351	1.0	2.6	8.4	0.15	-0.87	-0.25	-1.04	-0.28	-1.17	-0.24	-0.97	partiton by age, not by sex
P			<45		○	420	1.0	2.4	7.0		201	1.0	2.4	7.0		215	1.0	2.2	7.4	0.06	-0.27							
P			≥45		○	307	1.5	3.3	8.7		136	1.5	3.3	8.7		173	1.4	3.3	9.2	0.03	-0.28							
P	CEA	μg/L	All			667	0.4	1.3	4.3		316	0.5	1.6	4.2		351	0.4	1.1	4.2	0.09	-0.03							partiton by age for female
P			All	Smoker		567	0.4	1.2	3.9	○	238	0.5	1.4	3.8		289	0.4	1.1	4.0	0.13	-0.17	-0.16	-1.37	-0.14	-0.47	-0.15	-2.02	
P			<45	Smoker		315	0.4	1.1	3.3		135	0.4	1.3	3.5	○	180	0.3	1.0	3.3	0.15	0.21							
P			≥45	Smoker		193	0.5	1.4	4.6		81	0.6	1.5	3.9	○	151	0.5	1.4	5.2	0.09	-1.26							
P	CA19-9	kIU/L	All	Lewis(-)	○	639	2.3	5.7	29.3		301	1.9	5.0	25.6		345	2.4	6.4	26.8	-0.08	-0.17							
P	CA125	kIU/L	All			729	4.3	11.3	32.7	○	340	3.9	10.0	27.5	○	392	4.9	12.4	38.7	-0.13	-1.54	-0.08	-0.58	-0.17	-0.85	0.10	-0.38	partiton by sge for females
P			<45			420	3.9	12.1	30.5		202	3.5	10.1	25.2		218	5.4	13.9	37.0	-0.29	-1.75							
P			≥45			308	4.5	10.3	34.7		137	4.5	9.9	30.3		173	4.5	10.6	40.3	0.00	-1.30							
P	CA15-3	kIU/L	All		○	728	3.8	10.9	21.3		314	4.7	11.4	23.2		351	4.1	9.8	21.1	0.14	0.48							
P	Insulin	mIU/L	All			665	2.1	5.2	15.7		310	2.3	5.6	17.4		351	2.1	4.9	14.4	0.06	0.85	-0.043	-1.492	-0.054	-1.699	-0.025	-1.529	Need BMI restriction
P			All	BMI≥28	○	503	2.0	4.4	10.5		209	2.1	4.7	10.8		257	2.0	4.2	9.6	0.04	0.56							
P	Cortisol	nmol/l	All		○	736	162	337	606		353	187	366	596		353	154	313	620	0.29	-0.21							
P	GH	μg/L	All			636	0.0	0.8	4.5		316	0.00	0.05	0.48		354	0.01	0.8	12.4	-0.01	-10.5	0	0.6607	0	-0.245	0.0126	0.3254	Gaussian transform failed, need NP method
P			All	BMI≥28		465	0.0	1.2	5.2		201	0.00	0.06	0.45		259	0.05	1.1	13.5	-0.04	-9.8							
NP			All			670	0.0	0.2	7.3	○	341	0.01	0.04	2.99	○	396	0.04	0.8	7.9	-0.02	-2.6	0	0.523	0	-0.982	0.0035	0.3719	
NP			All	BMI≥28		470	0.0	0.3	8.3		211	0.01	0.05	2.24		259	0.05	1.3	8.6	-0.02	-3.0							
P	PRL	μg/L	All							○	340	3.5	7.5	16.3		338	3.7	9.6	27.3							0.20	2.31	partiton by age for female
P			PreMP	OC, TβhCG ≥2.9											○	242	4.3	10.8	30.0									
P			PostMP												○	118	3.1	6.5	16.1									
P	LH	IU/L	All	OC						○	336	1.4	3.2	8.1		338	1.7	9.0	78							-0.33	-0.95	partiton by age for female
P			PreMP	OC, TβhCG ≥2.9											○	241	2.0	6.7	42									
P			PostMP												○	117	8.4	28.4	61									
P	FSH	IU/L	<45							○	203	1.3	3.5	9.5														Obvious sex, age differences
P			≥45							○	136	2.4	5.2	20.2														
P			PreMP	OC, TβhCG ≥2.9											○	237	2.2	6.1	27.3									
P			PostMP												○	118	21	73	138									
P	TβhCG	IU/L	PreMP	OC											○	222	0.11	0.54	1.84									
P			PostMP												○	109	0.90	3.04	8.20									
P	Estradiol	pmol/L	All							○	339	6.8	72	175		344	3.6	171	2216							0.07	2.35	Gaussian transform failed in females
P			PreMP	OC, TβhCG ≥2.9											○	245	17	310	1519									
P			PostMP													122	0.0	28	212									
NP			All								315	3.7	73	185		344	3.7	204	1537							0.08	2.76	
NP			PreMP	OC, TβhCG ≥2.9												240	33	334	1499									
NP			PostMP												○	118	4.0	29	466									

			All=18–64		Males + Females					Males					Females					BR-Sex		BR-age MF (BRbmi MF)		BR-age M (BRbmi M)		BR-age F (BRbmi F)		Decision
Stat	Item	Units	Age	Other excl criteria		n	LL	Me	UL		n	LL	Me	UL		n	LL	Me	UL	LL	UL	LL	UL	LL	UL	LL	UL	
P	Progesterone	nmol/L	<45							○	204	**0.44**	**2.09**	**5.28**														Gaussian transform failed in females
P			≥45							○	135	**0.39**	**1.46**	**4.10**														
P			PreMP	OC, TβhCG ≥2.9												245	0.15	5.0	91									
P			PostMP												○	116	**0.10**	**0.84**	**3.36**									
NP			All								314	0.38	1.91	5.12		344	0.22	2.51	52.6							0.08	**2.76**	
NP			PreMP	OC, TβhCG ≥2.9											○	245	**0.34**	**3.7**	**55**									
NP			PostMP													118	0.04	0.80	4.7									
P	Testosterone	nmol/L	All							○	338	**6.9**	**12.3**	**22.5**		338	0.31	1.35	2.7					0.11	-0.02	-0.056	0.168	No need for BMI restriction
P			All	BMI≥28							211	7.3	13.8	22.5		248	0.28	1.35	2.8									
P			<45								204	6.9	13.1	22.6	○	200	**0.46**	**1.56**	**2.96**					0.29	0.00	-0.068	0.0411	
P			<45	BMI≥28							149	8.1	14.5	22.6		177	0.42	1.49	3.0									
P			≥45								133	6.7	11.4	21.6	○	171	**0.19**	**1.03**	**2.17**					0.06	**0.40**	-0.107	0.0319	
P			≥45	BMI≥28							79	6.9	12.1	23.1		90	0.14	0.94	2.2									
P	SHBG	nmol/L	All							○	293	**11**	**31**	**74**										0.10	-0.24			No need for BMI restriction
P			All	BMI≥28							176	13	33	70														
P			<45								164	11	28	65										0.18	0.26			
P			<45	BMI≥28							118	14	31	69														
P			≥45								125	13	37	82										-0.06	0.19			
P			≥45	BMI≥28							72	12	41	86														
P	PTH	ng/L	All		○	732	**19**	**39**	**74**		312	18	37	74		350	21	39	74	-0.18	0.05							
P	TSH	mU/L	All	TPO≥10	○	599	**0.64**	**1.59**	**3.75**		292	0.69	1.6	3.8		270	0.62	1.6	3.6	0.09	0.19							
P	FT4	pmol/L	All	TPO≥10	○	598	**8.4**	**11.1**	**14.2**		295	8.8	11.2	14.7		273	8.4	11.1	14.2	0.26	0.35							
P	FT3	pmol/L	All	TPOAb≥9 or TgAb≥4		547	4.1	5.1	6.4	○	220	**4.4**	**5.3**	**6.2**	○	211	**4.1**	**4.9**	**6.1**	0.36	0.10							
P	TT4	nmol/l	All	TPO≥10	○	567	**67**	**93**	**127**		283	65	91	122		282	70	94	132	-0.30	**-0.65**							
P	TT3	nmol/l	All	TPO≥10	○	561	**1.27**	**1.62**	**2.06**		282	1.29	1.65	2.01		280	1.26	1.59	2.14	0.17	**-0.68**							

PreMP = premenopausal PostMP = postmenopausal OC=oral contraceptives
