# Supplementary material for: Sources of variation and establishment of Russian reference intervals for major hormones and tumor markers
Source: PLoS One. 2021 Jan 7;16(1):e0234284. doi: 10.1371/journal.pone.0234284 (PMC7790266; doi:10.1371/journal.pone.0234284)
Supplement: Attachment — Submitted filename: reviewer comments 1.pdf [file pone.0234284.s009.pdf]

Editor in Chief, PLOS ONE

Sources of variation and establishment of Russian reference intervals for major Hormones and tumor markers

PONE-D-20-15086

Reviewer(s)' Comments to Author:

Reviewer:

Comments to the Author
The manuscript describes the reference interval for most common immunoassays and discuss the sources of variation. The study is part of the global reference interval study of the IFCC.
The practical message of the manuscript is relevant and the statistical elaboration of data is very detailed. Some core aspects have weakened the manuscript.

Specific comments

Manuscript:
Introduction

- Page 4 Line 5-14: the listed information in this paragraph is repeated under materials and methods. The author can describe briefly the main outcomes of the previous study only.

Material and methods

Source data and target analytes

- Page 5: What are the males and females percentages in each region?
- Source and anthropometric data to be described by creating a new table (total and categorized subjects distribution between different regions, males and female counts, sex percentages, BP, BMI etc.).
- All described tests involved in the study can be summarized by creating a new table showing: full name of each test, its abbreviation, unit, and principle of measurement.
- How the new assays for both TSH and TβhCG are different from the old assays? i.e. Are they new generation, new lots, new components or antibodies…? etc.

Quality Control

- Page 5: The CV value for each test and the allowable limits described in the Westgard Website are to be listed in the previous suggested new table which contain tests names.

Results:

Miscellaneous hormones

- Page 11: excluding subjects with BMI≥28 is not clearly justified. It is well known that insulin is highly associated with BMI. Therefore, it is not convincing that the UL insulin RI for Russians is smaller than Asians with lower BMI (page14). In the first paper for Russian chemistry analytes RIs the following was described: "*The proportion of recruited obese volunteers with BMI>28 was 34% in males (127 persons) and 30% in females (125 persons)"*. Therefore, these percentages are not small enough to be ignored in the statistical analysis of the derived RI for insulin.

The justification needs to be discussed under the light of BMI prevalence in Russia (1st paragraph page 14) and to be supported by the following data in the section of results:

a- Insulin RI categorized by the BMI
b- Insulin RI calculated by NP method
c- Insulin RI without excluding subjects with BMI≥28.

- Page 11:
  Derivation of RIs:
  It seems the written paragraph under this title (last paragraph page 11) is truncated.
  "The first column stands for distinction between parametric (P) method and nonparametric (NP) method for RI derivation, the fourth column for……?"

- Page 13 Line 4-10: this paragraph is not clearly understood. It needs to be reworded.

- Table 4 (Part 1): the column of Russian study to be corrected to Current Russian study.

- Table 4 (Part 2): Chinese studies*. What does the asterisk mean?

- Page 17:
  Distinction between RIs and CDLs
  "For CA15-3 and CA19-9, our ULs are lower than commonly used cutoff values: 21.3 vs. 23.5KIU/l, and 29.3 vs. 35 KIU/l, respectively. The value between the ULs and cutoff values may be regarded as a gray zone for early detection of adenocarcinoma, although an increased false-positive rate is a problem in prioritizing the ULs."
  The author needs to clarify what is the point of getting RIs for Tumor Markers as long as the rate of false-positive results will be increased if ULs is adapted.

- Page 17: The full names for the abbreviation ISSAM, ISSM and TRT are required.
